# Bax-PGAM5L-Drp1 complex is required for intrinsic apoptosis execution

**DOI:** 10.18632/oncotarget.5013

**Published:** 2015-08-31

**Authors:** Wenjuan Xu, Linlin Jing, Quanshi Wang, Chung-Chih Lin, Xiaoting Chen, Jianxin Diao, Yuanliang Liu, Xuegang Sun

**Affiliations:** ^1^ Nanfang Hospital, Southern Medical University, Guangzhou, China; ^2^ School of Traditional Chinese Medicine, Southern Medical University, Guangzhou, China; ^3^ TCM Integrated Hospital of Southern Medical University, Guangzhou, China; ^4^ Department of Life Sciences and Institute of Genome Sciences, National Yang-Ming University, Taipei, Taiwan; ^5^ Zhujiang Hospital, Southern Medical University, Guangzhou, China

**Keywords:** PGAM5L, Bax, DRP1, intrinsic apoptosis, cancer

## Abstract

Intrinsic apoptosis eliminates cells with damaged DNA and cells with dysregulated expression of oncogene. PGAM5, a member of the phosphoglycerate mutase family, has two splicing variants: PGAM5L (the long form) and PGAM5S (the short form). It has been well established that PGAM5 is at the convergent point of multiple necrosis pathways. However, the role of PGAM5 in intrinsic apoptosis is still controversial. Here we report that the PGAM5L, but not PGAM5S is a prerequisite for the activation of Bax and dephosphorylation of Drp1 in arenobufagin and staurosporine induced intrinsic apoptosis. Knockdown of PGAM5L inhibits the translocation of Bax to the mitochondria and reduces mitochondrial fission. The interaction between PGAM5L and Drp1 was observed in both arenobufagin and staurosporine treated HCT116 cells, but not in HCT116 Bax^−/−^ cells. Bax transfection rescues the formation of the triplex in both arenobufagin and staurosporine stimulated HCT116 Bax^−/−^ cells. Arenobufagin shows remarkable anti-cancer effects both in orthotropic and heterotropic CRC models and demonstrates less toxic effects as compared with that of cisplatin. Bax-PGAM5L-Drp1 complex is detected in arenobufagin and staurosporine treated CRC cells *in vitro* and in arenobufagin and cisplatin treated tumor *in vivo* as well. In summary, our results demonstrate that Bax-PGAM5L-Drp1 complex is required for intrinsic apoptosis execution.

## INTRODUCTION

Cancer is a major public health problem which accounts for 1/4 deaths in the United States [1]. Apoptosis resistance is a strategy of choice for cancer cells that employ multiple mechanisms to override apoptosis for survival [2]. Understanding and tweaking the mechanisms of apoptosis are pivotal to eliminate potentially malignant cells.

PGAM5, a member of the phosphoglycerate mutase family, is a Kelch-like ECH-associated protein 1 (Keap1)-binding protein. PGAM5 has two splicing forms, the shorter form (PGAM5S) and the longer isoform (PGAM5L). Both isoforms of PGAM5 function in the intrinsic necrosis pathway, based on the evidence that PGAM5 knockdown attenuates ROS- and calcium ionophore induced necrotic death [3]. The “mitochondria attack complex” consisting of PGAM5 and Drp-1 is formed during the execution of necrosis [4]. However, the role of PGAM5 in apoptosis is still controversial. Staurosporine-induced apoptotic death is not affected by PGAM5 knockdown.

Upon apoptotic stimuli, Bax, Bak, and possibly other unidentified proteins are relocated into the mitochondrial outer membrane and oligomerized to form the mitochondrial apoptosis-induced channel to release cyto *C* [5]. Arenobufagin [6] and staurosporine [7, 8] have been reported to induce apoptosis in different cell lines through activation of Bax. Thus we want to examine if PGAM5 is necessary in Bax mediated apoptosis. Our results identify a multiprotein complex including PGAM5, Bax and Drp1 that specifically formed during intrinsic apoptosis induction.

## RESULTS

### Arenobufagin induces tumor cell apoptosis

**Figure 1:**
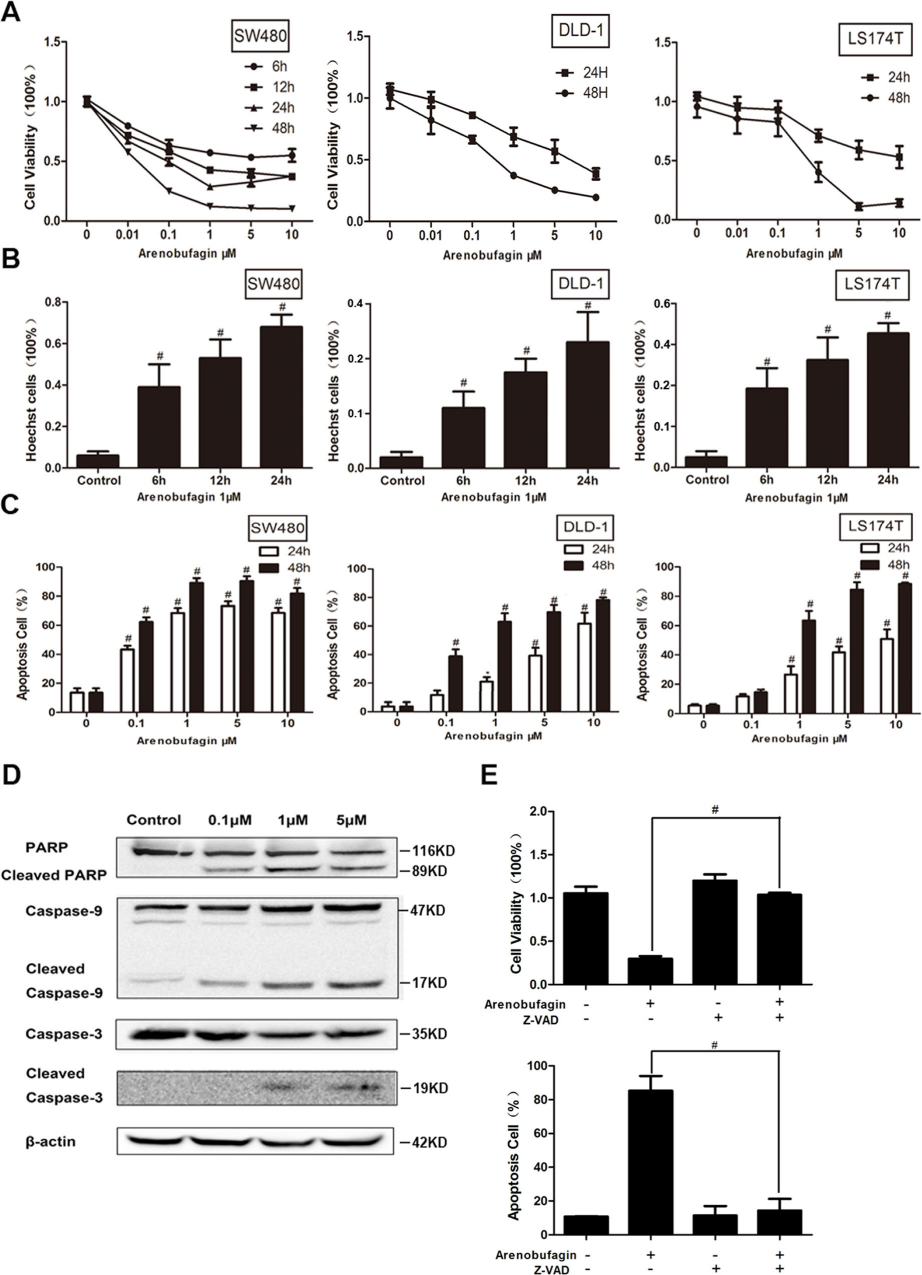
Arenobufagin induces tumor cell apoptosis **A.** The percentage of viable cells measured by the MTT assay at 24 and 48 h relative to no-drug controls and arenobufagin concentrations were plotted as log-dose response curve (*n* = 6 per group). **B.** Cells were stained with Hoechst33342 (5 μg/ml) and subjected to analysis of apoptosis population (*n* = 3). **C.** PE-annexin V/7-amino-actinomycin D exposed on the cell surface was measured by flow cytometric analysis (*n* = 3). Data derived from three separate experiments are presented as the means ± S.E.M. **D.** Total cell lysates were prepared for western blot analysis of the apoptosis regulatory proteins (*n* = 3). **E.** Cells were treated with the combination of arenobufagin and Z-VAD-fmk (20 μM). Cell viability was assayed or annexin V-positive cells were quantitatively analyzed (*n* = 6 per group). *P < 0.05, ^#^*P* < 0.01, one-way ANOVA, post hoc comparisons, Tukey's test. Columns, means; error bars, SEs. See also Supplementary Figure S1.

To address the role of arenobufagin on cell viability, various CRC cell lines, including SW480, DLD-1 and LS174T, were tested. Arenobufagin decreased cell viability both in a dose - and time - dependent manner (Figure 1A–1B). Arenobufagin also lowered the cell viability in HeLa (human cervical cancer cell line), A549 (human lung adenocarcinoma epithelial cell line), MCF-7 (human breast adenocarcinoma cell line), and even in taxol resistant MCF-7/taxol cell line (Supplementary Figure S1C). We then examined which cell death subroutine was responsible for the lowered viability. Rounding-up of the cells, retraction of pseudopodes, reduction of cellular and nuclear volume (pyknosis) and nuclear fragmentation (karyorrhexis) in arenobufagin treated SW480 cells suggested the morphological features of apoptosis [9] (Supplementary Figure S1B). Hoechst 33342 staining (Supplementary Figure S1A), annexin V/7-amino-actinomycin D double staining (Figure 1C and Supplementary Figure S1D) showed that most of the cell death induced by arenobufagin can be classified as apoptosis in SW480, DLD-1, Hela, and A549.

Activation of caspases is a biochemical feature of apoptosis [9]. Immunoblotting assessment showed that caspase 9 was cleaved by arenobufagin. Activated caspase-9 in turn cleaves and activates caspase-3. The cleaved caspase 9 and caspase 3 were increased by arenobufagin in a dose-dependent manner. The cleavage of poly (ADP) ribose polymerase (PARP), a caspase-3/7 substrate [10], was also increased by arenobufagin treatment (Figure 1D). The apoptosis caused by arenobufagin was efficiently abrogated by pretreatment with N-benzyloxycarbonyl -Val-Ala-Asp-fluoromethylketone (Z-VAD-fmk), a broad spectrum caspase inhibitor, suggesting that arenobufagin induced cell death was caspase-dependent [9]. The viabilities were subsequently recovered as showed in Figure 1E. These morphological and biochemical changes suggest that the cell death caused by arenobufagin is apoptosis.

### The intrinsic apoptosis caused by arenobufagin is Bax-dependent

**Figure 2:**
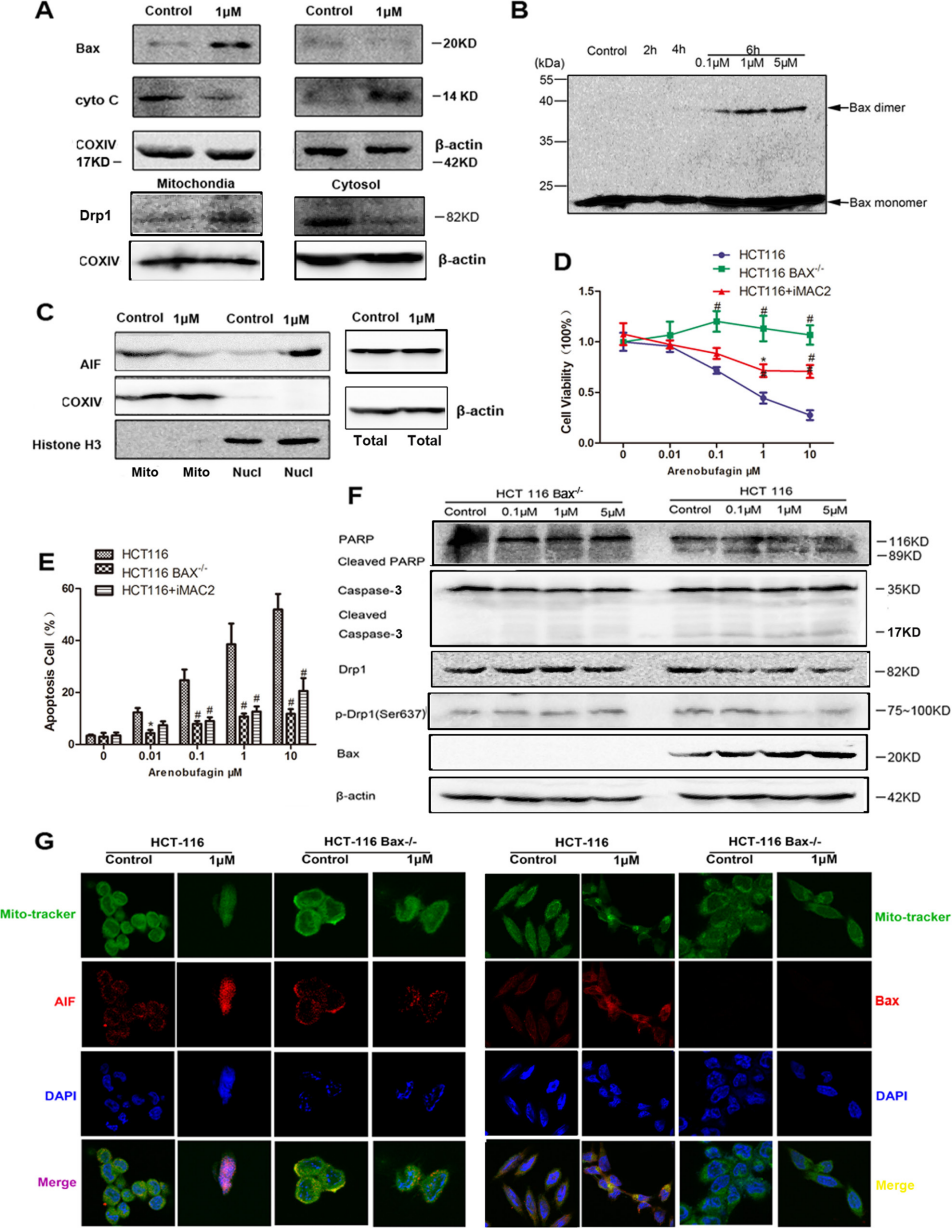
The intrinsic apoptosis caused by arenobufagin is Bax-dependent **A.** Level of Bax, cyto *C*, and Drp1 in cytosol and mitochondrial fractions from HCT116 WT cells treated with arenobufagin at the indicated concentrations for 24 h were evaluated by western blot analysis (*n* = 3). β-actin and COXIV were used as a loading control, respectively. **B.** HCT116 WT cells were incubated with the crosslinker (disuccinimidyl suberate) and subjected to western blot analysis using a Bax antibody. **C.** Mitochondrial and nuclear fractions separated from were loaded and immunoblotted with AIF. The expression of AIF was normalized against the level of corresponding Histone H3 (*n* = 3). HCT116 WT Cells were pretreated with iMAC2 (20 μM) for 3 h prior to a 24 h treatment with arenobufagin. HCT116 WT+iMAC2, HCT116 WT and HCT116 Bax^−/−^ cells viability was measured by MTT assay **D.** and the proportion of annexin V-positive cells was analyzed by flow cytometry **E.** (*n* = 6 per group). **F.** Western blotting experiments for PARP, Caspase-3, Drp1, p-Drp1, and Bax were performed with the cell lysates obtained after arenobufagin treatment (*n* = 3). **G.** Cells were labeled with MitoTracker and immunostained using AIF and Bax antibody followed by labeling with Cy3 and counterstaining with DAPI (*n* = 3). Representative immunofluorescence images are shown.1000× for all, scale bar = 10 μm. See also Supplementary Figure S2.

Arenobufagin induced translocation of Bax to the mitochondria was found in a dose-dependent manner (Supplementary Figure S2A). Moreover, the translocation and accumulation of Bax and Drp1 in the mitochondria were observed in HCT116 WT cells (Figure 2A). Dimers were formed when cells were treated with arenobufagin (Figure 2B). Given the robust MOMP activity of Bax activation, we therefore assayed its ability to release soluble pro-apoptotic factors liked cyto *C* and non-soluble factors, such as apoptosis-inducing factor (AIF) that tethered to the outer surface of the inner mitochondrial membrane (IMM) [5]. Release of cyto *C* into cytosol (Figure 2A) and translocation of AIF from mitochondria to nucleus were observed in HCT116 WT cells (Figure 2C).

The role of Bax in arenobufagin induced apoptosis was further confirmed with HCT116 WT and HCT116 Bax^−/−^ cells. Arenobufagin significantly increased the apoptosis rate and decreased the cell viability in HCT116 WT cells compared with that of HCT116 Bax^−/−^ cells in a dose dependent manner. The increased apoptosis and decreased viability could be partly reversed by iMAC2, a potential inhibitor of mitochondrial apoptosis-induced channel (Figure 2D–2E) [11]. Cleavage of PARP and caspase-3 were observed in HCT116 WT cells accompanied by Bax activation (Figure 2F). JC-1 is a reliable fluorescent probe to assess ΔΨm [12]. The concentration-dependent shift in the emission spectrum from red to green measured by flow cytometry indicated the dissipation of Δ*Ψ*m by arenobufagin in SW480 and DLD-1 cells (Supplementary Figure S2B and Supplementary Figure S2D). The lowered ΔΨm by arenobufagin was significantly reversed in HCT116 Bax^−/−^ cells (Supplementary Figure S2C and S2D).

Immunofluorescent staining provided direct evidences that arenobufagin drived AIF shifting to the nucleus with Bax punctae in the mitochondria (Figure 2G). It has been shown that both MOMP and proteolytic cleavage of mature AIF from its IMM-embedded N-terminus were prerequisites for AIF release [13]. This suggested that not only arenobufagin has a strong permeabilization effect on mitochondrial membranes, but also has efficient effect in disrupting the inner mitochondrial membrane integrity [5]. According to the Nomenclature Committee on Cell Death [14], the dissipation of Ψm, the loss of cyto C from mitochondria and translocation of AIF to the nuclear, suggest that the cell death induced by arenobufagin can be defined as intrinsic apoptosis, which is highly dependent on Bax activation.

### PGAM5L is required for Bax-mediated intrinsic apoptosis

**Figure 3:**
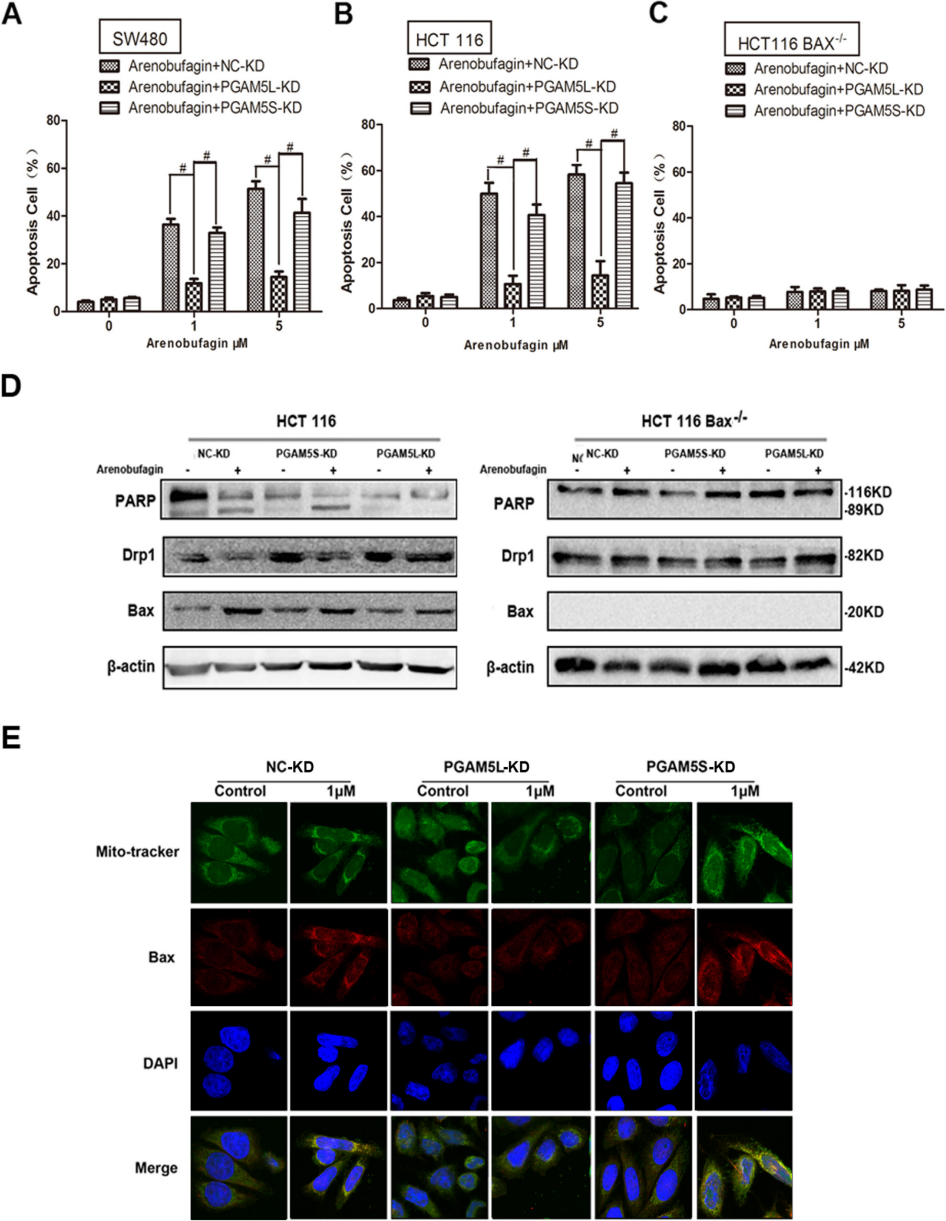
PGAM5L is required for Bax-mediated intrinsic apoptosis **A–C.** SW480, HCT116 WT and HCT116 Bax^−/−^ cells were treated with arenobufagin in the absence or presence of PGAM5L-KD and PGAM5S-KD. The proportion of annexin V-positive cells was analyzed by flow cytometry (*n* = 3). **D.** HCT-116 cells or HCT-116 Bax^−/−^ cells were transfected with PGAM5L-KD or PGAM5S-KD. Immunoblot analysis of PARP, Drp1 and Bax were determined after 24 h of arenobufagin treatment. **E.** Confocal immunofluorescence of Bax (red, anti-Bax) in the PGAM5L-KD and PGAM5S-KD SW480 cells that were loaded with MitoTracker and DAPI. 1000× for all, scale bar = 10 μm. See also Supplementary Figure S3.

The mitochondrial PGAM5 is at the convergent point of multiple necrosis pathways. Two splicing forms of PGAM5 are identical from amino acid 1–239, with the shorter form (PGAM5S) containing 16 additional C-terminal amino acids and the longer isoform (PGAM5L) containing 50 additional C-terminal amino acids. It should be noted that the two variants of PGAM5 carry out different functions during necrosis execution [3]. PGAM5S is more hydrophobic than PGAM5L. PGAM5S was only observed in the 1% SDS-soluble fraction (the Triton X-100 insoluble pellet with a buffer containing 1% SDS). Therefore, PGAM5S was not found in the whole-cell extraction (WCE) buffer but only with PGAM5L (containing 1% Triton X-100) [3]. To further confirm the discrepancy between PGAM5L and PGAM5S, His-tagged PGAM5S and Flag-tagged PGAM5L coding sequence were constructed and transfected into SW480 cells. As shown in Supplementary Figure S3B, His and Flag expressed only in the buffer of SDS-soluble and WCE respectively which were demonstrated by incubating with anti-His, anti-Flag, anti-PGAM5, and anti-vimentin antibodies. These results indicated that PGAM5S only presented in the 1% SDS-soluble fraction, and PGAM5L only presented in WCE buffer. The finding that PGAM5L and PGAM5S are in different extraction allowed us to dissect the roles of these two splice forms in apoptosis. Supplementary Figure S3C showed the specific knockdown efficacy of PGAM5L and PGAM5S isoforms by western blot. We then examined their role in arenobufagin induced intrinsic apoptosis. The apoptosis rate was significantly decreased in SW480 cells infected with lentivirus carrying short hairpin RNA (shRNA) of PGAM5L, but not of PGAM5S (Figure 3A, Supplementary Figure S3A) [3]. The reduced apoptosis in HCT116 cells stably transduced with lentivirus carrying shRNA of PGAM5L, but not of PGAM5S, further confirmed our results (Figure 3B). However, the apoptosis rate was less than 5% in arenobufagin treated HCT116 Bax^−/−^ cells stably expressing PGAM5L or PGAM5S shRNA (Figure 3C). Accordingly, the viability was elevated in SW480 cells infected with PGAM5L shRNA or in HCT116 PGAM5L shRNA stable cells, but not in PGAM5L shRNA HCT116 Bax^−/−^ cells treated with arenobufagin (Supplementary Figure S3D–S3F).

The cleavage of PARP was observed in PGAM5L shRNA transfected HCT116 cells, but not in HCT116 Bax^−/−^ cells (Figure 3D). We asked if PGAM5L is needed in the translocation of Bax. The overlap between mito-tracker (green) and Bax staining (red) which showed yellow fluorescence (merge) indicates that arenobufagin induced relocation of Bax to mitochondria in SW480 cells. PGAM5L-KD, but not PGAM5S -KD, inhibited the translocation of Bax induced by arenobufagin (Figure 3E). Taken together, our findings demonstrate that PGAM5L is required for arenobufagin induced intrinsic apoptosis mediated through Bax translocation to mitochondria.

### Drp1 is also needed in the induction of intrinsic apoptosis process

**Figure 4:**
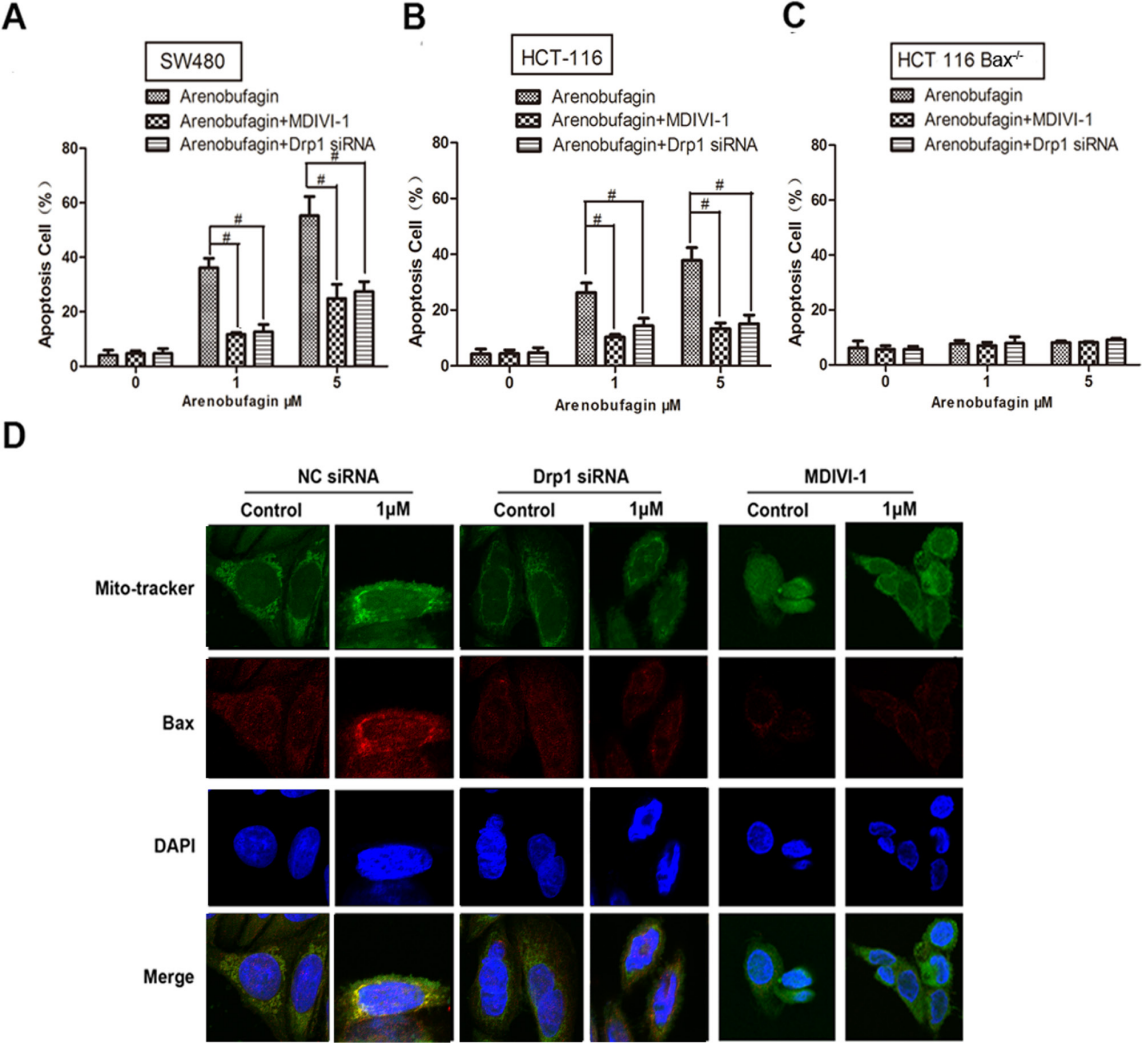
Drp1 is also needed in the induction of intrinsic apoptosis process **A–C.** SW480, HCT116 WT and HCT116 Bax^−/−^ cells were treated with arenobufagin in the absence or presence of Drp1 siRNA (100 nM) and Mdivi-1 (50 μM). The proportion of annexin V-positive cells was analyzed by flow cytometry (*n* = 3). **D.** Confocal immunofluorescence of Bax (red, anti-Bax) in the Drp1 siRNA and Mdivi-1 SW480 cells that were loaded with MitoTracker and DAPI (*n* = 3). 1000× for all, scale bar = 10 μm. See also Supplementary Figure S4.

Drp1 siRNA or Drp1 inhibitor mdivi-1 [15] significantly inhibited arenobufagin induced apoptosis in SW480 and HCT116 WT cells (Figure 4A–4B, Supplementary Figure S4A). Accordingly, Drp1 siRNA recovered the viability in both cell lines (Supplementary Figure S4B–S4C). Cell viability and apoptosis rate were not affected in HCT116 Bax^−/−^ cells with or without arenobufagin treatment, nor are they affected by PGAM5L siRNA or PGAM5S siRNA (Figure 4C, Supplementary Figure S4D).

The lowered expression of Drp1 by arenobufagin together with its activation of Bax were interfered by PGAM5L siRNA, but not by PGAM5S siRNA (Figure 3D). Immunofluorescence staining showed that the accumulation of Bax in mitochondria induced by arenobufagin was severely disrupted by Drp1 siRNA or mdivi-1 in SW480 cells (Figure 4D). Drp1 was reported to facilitate the membrane fission via constricting and severing the mitochondrial membrane through a GTP-dependent mechanism [16]. It is required for cytochrome *C* release and consequent activation of caspases during apoptosis [17]. We then checked the expression of Drp1 in CRC patient tissues and tissue microarrays. The expression of Drp1 in tumor was significantly stronger than that in tumor adjacent tissues and normal tissues (Supplementary Figure S4E). Microarray analysis further confirmed the above results that the expressions of Drp1 in malignant and adjacent tissues were higher than that of normal colon and normal lymphoid tissues (Supplementary Figure S4F, sample A–G). Thus, together our data suggest that Drp1 is an inseparable partner of PGAM5L in the induction of intrinsic apoptosis with Bax activation. Targeting Drp1-mediated mitochondrial fission might be an effective approach for treating cancer [18].

**Figure 5:**
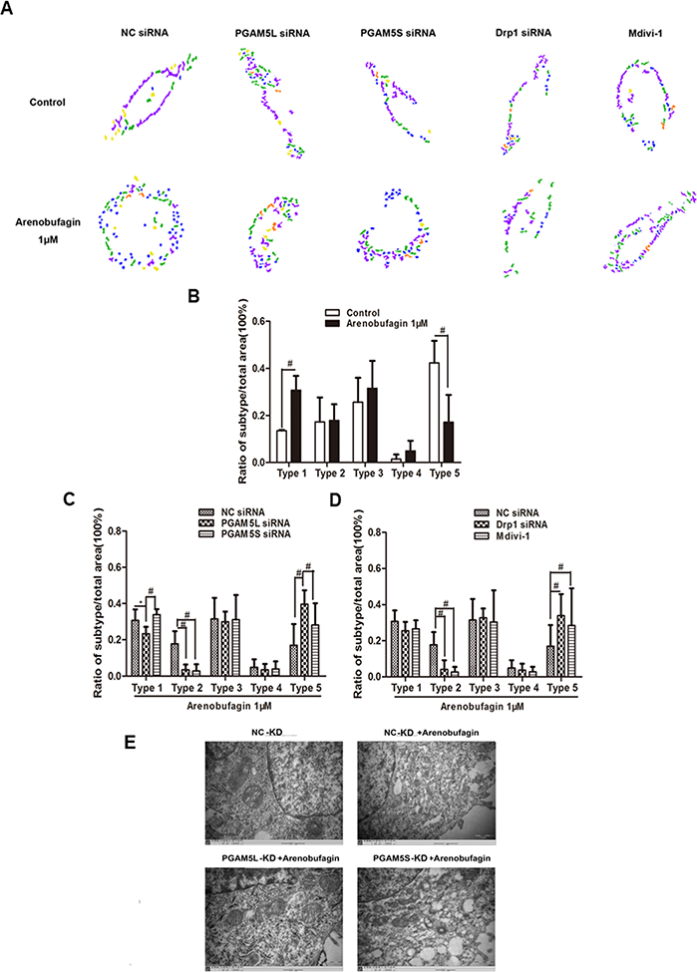
PGAM5 and Drp1 regulates mitochondrial morphology in the induction of apoptosis **A.** Micro-P analysis of mitochondrial morphology in HCT116 WT cells treated with arenobufagin in the absence or presence of PGAM5L siRNA, PGAM5S siRNA, Drp1 siRNA (100 nM) or Mdivi-1 (50 μM). The Micro-P algorithm classified mitochondria into small fragmented mitochondrion (type 1, blue), large fragmented mitochondrion (type 2, yellow), straight tubular mitochondrion (type 3, green), curved tubular mitochondrion (type 4, orange), and horse-shoe, donut, or network tubular mitochondrion (type 5, purple). **B.** A representative presentation of Micro-P analysis is shown. **C–D.** Bar graphs at the bottom show the percentages of type 1 to type 5 mitochondria, and are expressed as the means ± S.D. *P < 0.05, ^#^*P* < 0.01, *n* = 5, one-way ANOVA, post hoc comparisons, Tukey's test. Columns, means; error bars, SEs. **E.** Mitochondrial morphological changes of arenobufagin-treated cells as observed by TEM. 15000× for all, scale bar = 2 μm.

Downregulation of Drp1 inhibits fragmentation of the mitochondrial network and partially prevents the release of cyto *C* [19]. Mdivi-1 prevents mitochondria division and Bax-mediated mitochondrial outer membrane permeabilization during apoptosis [20]. We quantified PGAM5 and Drp1 mediated mitochondrial morphological changes using a recently developed software, Micro-P [21]. The number of small fragmented mitochondria (type 1) was significantly increased and the number of normal mitochondria was decreased in cell treated with arenobufagin (Figure 5A–5B). PGAM5L siRNA, but not PGAM5S siRNA alleviated the increasing of type 1 mitochondria induced by arenobufagin (Figure 5C). Drp1 siRNA and Mdivi-1 treatment displayed a significantly higher percentage of type 5 mitochondrion, with a concomitant decrease in type 2 (Figure 5D). Electron microscope images showed that the increased small fragmented mitochondria were significantly reduced by PGAM5L shRNA (Figure 5E). These results suggested that interaction between PGAM5L and Drp1 is linked to mitochondria fission.

### Bax interacts with PGAM5-Drp1 complex

**Figure 6:**
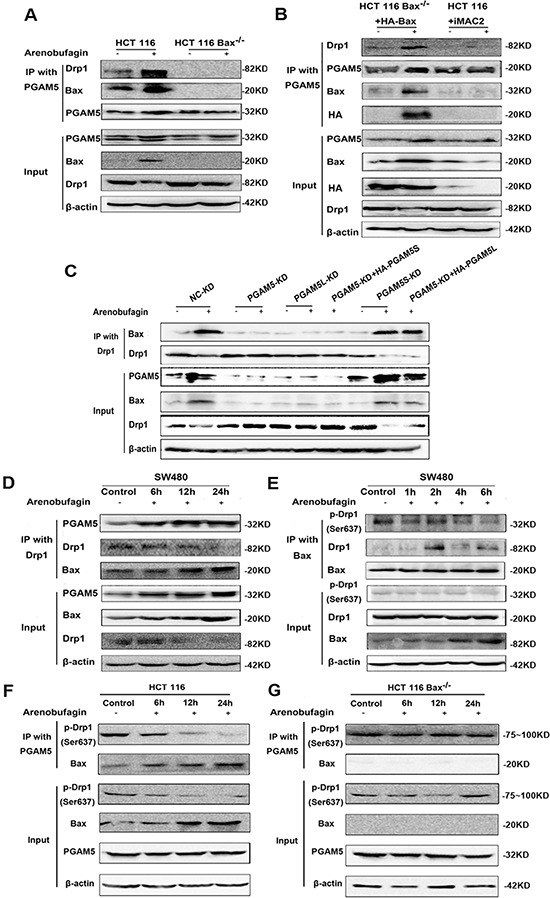
Bax Interacts with PGAM5-Drp1 Complex **A.** HCT116 WT and HCT116 Bax^−/−^ cells were treated with 1 μM arenobufagin for 12 h. IP was performed with an anti-PGAM5 antibody. Co-IP Drp1 and Bax were detected by western blotting (*n* = 3). **B.** HCT116 Bax^−/−^ cells were transfected with the control vector or HA-Bax, and treated with 1 μM arenobufagin for 12 h. HCT116 WT Cells treated with the combination of arenobufagin and iMAC2 (20 μM). Cells were lysed and immunoprecipitated using an anti-PGAM5 antibody. Immunoprecipitates were subjected to western blotting using Drp1 and Bax antibody (*n* = 3). **C.** PGAM5-KD HCT116 WT cells were transfected with PGAM5L or PGAM5S coding sequence, and the cells were then treated with arenobufagin for 12 h. IP was performed with a PGAM5 or Drp1 antibody. Co-IP PGAM5, Drp1 and Bax was detected through western blotting (*n* = 3). **D.** SW480 cells were treated with 1 μM arenobufagin for different time, and then IP was performed with Drp1 antibody; Co-IP PGAM5, Drp1 and Bax was detected by western blotting (*n* = 3). **E.** SW480 cells were treated with 1 μM arenobufagin for different time, and then IP was performed with Bax antibody; Co-IP p- Drp1 (ser 637), Drp1 and Bax was detected by western blotting (*n* = 3). **F–G.** HCT116 WT and HCT116 Bax^−/−^ cells were treated with 1 μM arenobufagin for different time. IP was performed with an anti-PGAM5 antibody. Co-IP p- Drp1 (ser 637) was detected by western blotting (*n* = 3).

Once the “mitochondria attack complex” [3] of PGAM5 and Drp-1 is formed, dimerization and activation of Drp1 would lead to mitochondrial dysfunction and cell necrosis [4]. To determine if the specific association between Drp1 and PGAM5 is required for the induction of intrinsic apoptosis, co-immunoprecipitation was performed in HCT116 WT and HCT116 Bax^−/−^ cells. As shown in Figure 6A, a strong interaction between Bax, Drp1 and PGAM5 was found in HCT116 WT under apoptotic condition when proteins were immunoprecipitated with PGAM5 antibody. The interaction was completely abolished in HCT116 Bax^−/−^ cells under both normal and apoptotic conditions. To further confirm the results, we tried to rescue apoptosis by transfecting a HA-tagged Bax coding sequence into HCT116 Bax^−/−^ cells. As expected, the interaction between Bax, Drp1 and PGAM5 was restored. Also, iMAC2, a potential inhibitor of mitochondrial apoptosis-induced channel [11], attenuated the interaction between Drp1 and PGAM5 in apoptotic HCT116 WT cells (Figure 6B). These results suggest that a Drp1-PGAM5 complex is formed in a Bax dependent manner.

Recent study demonstrated that mitochondrial fragmentation facilitates Bax insertion and activation in mitochondria, resulting in the release of apoptogenic factors, such as cyto *C*. Drp1 inhibition blocks Bax insertion and activation in mitochondrial membrane [22]. As shown in Figure 6C, PGAM5L shRNA, but not PGAM5S shRNA, almost completely abolished the interaction between Drp1 and Bax. When PGAM5L expression was restored, the interaction was re-established.

The interaction between PGAM5, Drp1 and Bax was confirmed in SW480, Hela and A549 cell lines (Supplementary Figure S1E). The immunoprecipitated Bax and PGAM5 increased in a time dependent manner as baited with Drp1 under arenobufagin treatment in SW480 (Figure 6D). The dephosphorylation of Drp1 and Bax at early time points after arenobufagin exposure was explored that dephosphorylation of Drp1 Ser637 was observed in the first 1, 2, 4, 6 hours and the strongest interaction between Drp1 and Bax was observed after 2 hrs treatment (Figure 6E).

A time dependent dephosphorylation of Drp1 Ser637 was observed in arenobufagin treated HCT116 WT cells, but not in HCT116 Bax^−/−^ cells. The dephosphorylation of Drp1 Ser637 was positively correlated with the expression of Bax (Figure 6F–6G).

These results further strengthen our results that Bax activation and Drp1 dephosphorylation are indispensable for the formation of the Bax-PGAM5L-Drp1 complex in the process of intrinsic apoptosis.

### Staurosporine induces the formation of the Bax-PGAM5L-Drp1 complex

**Figure 7:**
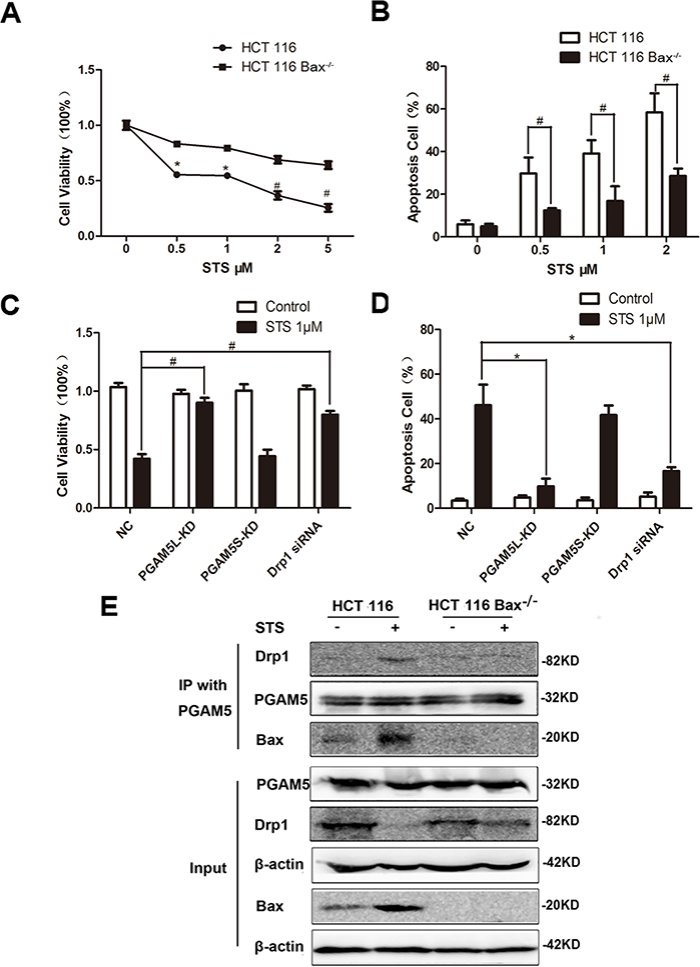
Staurosporine induces the formation of the Bax-PGAM5L-Drp1 complex HCT116 WT and HCT116 Bax^−/−^ cells viability was measured by MTT assay (*n* = 6) **A.**, and the proportion of annexin V-positive cells was analyzed by flow cytometry (*n* = 3) **B.** Cells were treated with 1 μM STS in the absence or presence of PGAM5L-KD, PGAM5S-KD and Drp1 siRNA. Cell viability was measured by MTT assay (*n* = 6) **C.**, and the proportion of annexin V-positive cells was analyzed by flow cytometry (*n* = 3) **D. E.** HCT116 WT and HCT116 Bax^−/−^ cells were treated with 1 μM arenobufagin. IP was performed with an anti-PGAM5 antibody. Co-IP Drp1 and Bax was detected by western blotting (*n* = 3).

To eliminate the possibility that the Bax-PGAM5L-Drp1 complex was only formed in the presence of arenobufagin, staurosporine was selected to re-examine if the interactions among Bax, PGAM5L and Drp1 were indispensable for intrinsic apoptosis execution. The viability in HCT116 WT cells was significantly lower than that of HCT116 Bax^−/−^ cells under the treatment of staurosporine (Figure 7A). Annexin V staining showed that the apoptosis rates in HCT116 WT cells were significantly higher than that of HCT116 Bax^−/−^ cells, suggesting that staurosporine induced apoptosis partially depended on Bax activation [7] (Figure 7B). PGAM5L shRNA and Drp1 siRNA, but not PGAM5S shRNA, significantly elevated the viability of SW480 cells treated with 1 μM staurosporine (Figure 7C). The lower apoptosis rates induced by silencing PGAM5L and Drp1 might be responsible for the elevated viability (Figure 7D). Immunoprecipitation data showed that the interaction between PGAM5 and Drp1 highly depended on activation of Bax (Figure 7E, lane 2) in HCT116 WT cells, but not in HCT116 Bax^−/−^ cells, further confirmed that each component of the Bax-PGAM5L-Drp1 complex is necessary for the induction of intrinsic apoptosis.

### Arenobufagin inhibits growth and metastasis of orthotopically implanted colorectal carcinoma through inducing apoptosis

**Figure 8:**
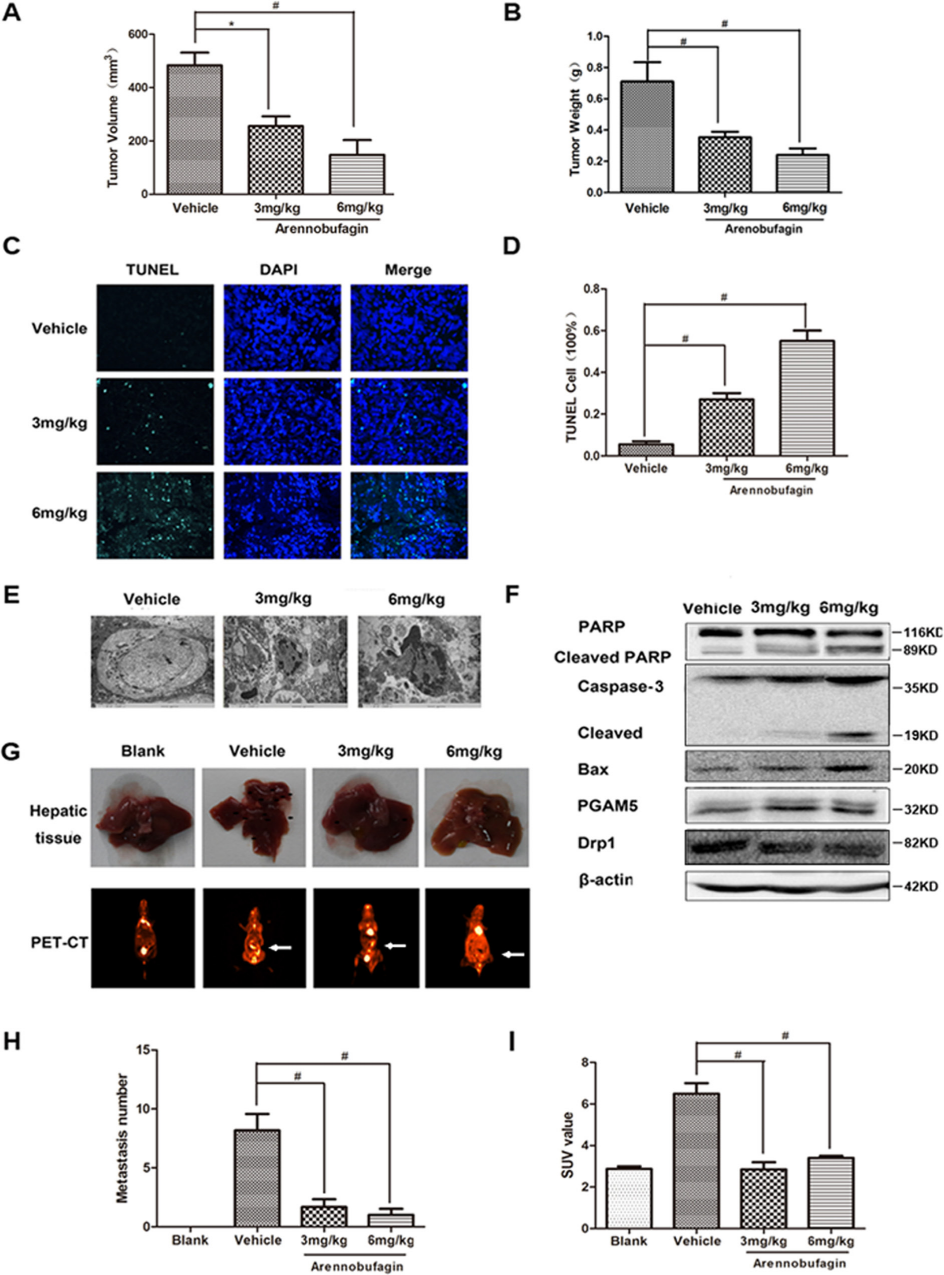
Arenobufagin inhibits growth and metastasis of orthotopically implanted colorectal carcinoma through inducing apoptosis **A.** Isolated tumor size and **B.** Tumor weight from the SW480-eGFP mouse Orthotopic CRC model. **C–D.** Tumors were excised and processed for immunostaining with TUNEL Kit(green) and DAPI (blue), and fluorescent images were obtained by microscopy, 400× for all, scale bar = 150 μm. **E.** Morphological changes of arenobufagin-treated tumors as observed by TEM. 15000× for all, scale bar = 2 μm. **F.** PARP, Caspase-3, Drp1, PGAM5 and Bax in tumor tissue lysates from vehicle- and arenobufagin-treated mice were detected by western blot analysis. **G.** The image of metastasis in mice hepatic tissue (above) and PET-CT images (below). **H.** Bar graphs at the bottom show the percentages of metastasis number in mice hepatic tissue. **I.** Ratio of 18F-FDG SUV of drug treatment versus control in whole body for PET-CT mice. *P < 0.05, ^#^*P* < 0.01, *n* = 6, one-way ANOVA, post hoc comparisons, Tukey's test. Columns, means; error bars, SEs. See also Supplementary Figure S5 and Supplementary Video S1–S4.

To characterize the role of arenobufagin in apoptosis, colorectal carcinomas were orthotopically implanted into BALB/c-nu mice as described previously [23]. Arenobufagin decreased the tumor volume *in situ* significantly (Figure 8A). Vernier caliper measurement showed that arenobufagin decreased the tumor size in a dose - dependent manner (Figure 8B, Supplementary Figure S5A). Increased TUNEL positive cells indicated that the cell death induced by arenobufagin could be due to apoptosis (Figure 8C–8D). The apoptotic bodies observed by electron microscope further confirmed that cell death induced by arenobufagin was apoptosis (Figure 8E). Western blot analysis showed that cleaved PARP and caspase 3 were increased significantly in arenobufagin treated mice (Figure 8F). The expression of Bax was elevated and Drp1 was decreased by arenobufagin treatment (Figure 8F and Supplementary Figure S5B). Visual inspection and *in vivo* imaging with positron emission computed tomography provided direct evidences that arenobufagin decreased lived metastasis of CRC (Figure 8G, and Supplementary Video S1–S4). The metastatic number and standardized uptake value (SUV) measured by fluorodeoxyglucose positron emission tomography were significantly reduced by arenobufagin (Figure 8H–8I). Collectively, these results demonstrated that arenobufagin inhibited the growth and metastasis of CRC by inducing Bax mediated apoptosis.

### Arenobufagin and cisplatin suppress heterotropic CRC growth in a Bax-dependent manner

**Figure 9:**
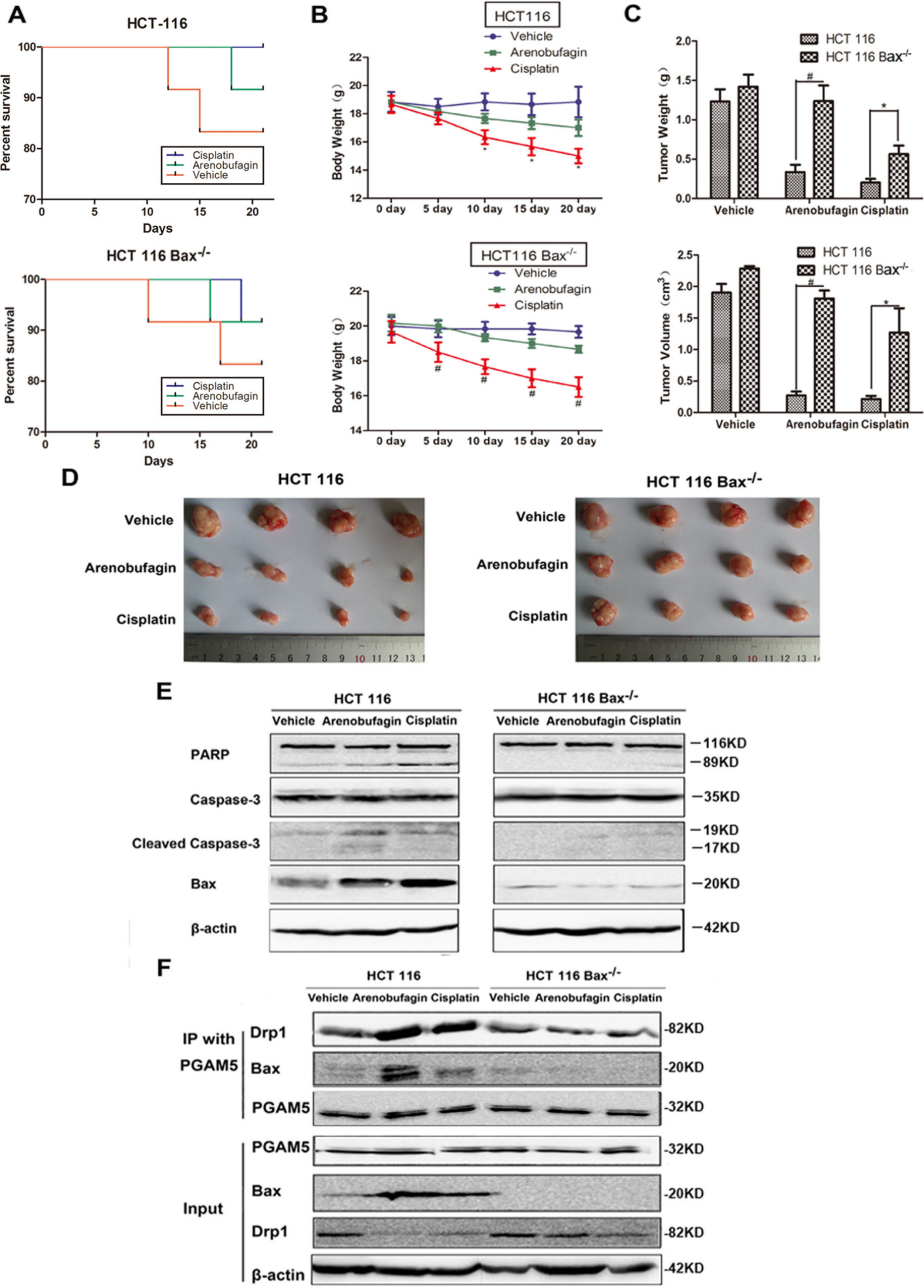
Arenobufagin and cisplatin suppress heterotropic CRC growth in a Bax-dependent manner **A.** Effect of Arenobufagin and Cisplatin on the survival ratio of mice. **B.** Effect of Arenobufagin and Cisplatin on body weight of mice. **C.** Isolated tumor size and tumor weight from the HCT116 WT and HCT116 Bax^−/−^ mice heterotropic CRC model. **D.** Microscopic view of colon tumor tissue in mice. **E.** PARP, Caspase-3, and Bax in tumor tissue lysates from vehicle-, arenobufagin-, and cisplatin-treated mice were detected by western blot analysis. **F.** HCT116 WT and HCT116 Bax^−/−^ mice were treated with arenobufagin and cisplatin. IP was performed with an anti-PGAM5 antibody. Co-IP Drp1 and Bax was detected by western blotting. *P < 0.05, ^#^*P* < 0.01, *n* = 12, one-way ANOVA, post hoc comparisons, Tukey's test. Columns, means; error bars, SEs. See also Supplementary Figure S6.

To further investigate if aenobufagin induced intrinsic apoptosis is Bax-dependent *in vivo*, heterotropic CRC tumors derived from HCT116 Bax^+/+^ and Bax^−/−^ cells were xenografted to BALB/c-nu mice. As staurosporine binds to many kinases with high affinity and low selectivity, it has been precluded in clinical use [24]. Cisplatin, a widely used chemotherapy drug which binds to DNA and triggers apoptosis, was chosen in the *in vivo* test. The death rate and body weight loss caused by arenobufagin are lower than that of caused by cisplatin in both HCT116 Bax^+/+^ and Bax^−/−^ tumor bearing mice (Figure 9A–9B). Arenobufagin decreased tumor weight (*P* < 0.01) and tumor volume (*P* < 0.01) of Bax^+/+^ tumor as compared to the Bax^−/−^ group. Cisplatin also decreased tumor weight (*P* < 0.05) and tumor volume (*P* < 0.05) of Bax^+/+^ tumor as compared to the Bax^−/−^ group (Figure 9C–9D and Supplementary Figure S6A). Cleaved PARP and caspase-3 were elevated in arenobufagin and cisplatin treated Bax^+/+^ mice but not in Bax^−/−^ mice (Figure 9E). When baited with PGAM5, Drp1 and Bax were immunoprecipitated from arenobufagin and cisplatin treated Bax^+/+^ tumor tissues extracts (Figure 9F). Bax activation and reduced Drp1 were observed as expected in arenobufagin and cisplatin treated Bax^+/+^ mice, but not Bax^−/−^ mice (Figure 9F and Supplementary Figure S6B). PGAM5S was only present in the 1% SDS-soluble fraction but not in our used Triton X-100 extraction buffer [3]. The *in vivo* formed complex might be Bax-PGAM5L-Drp1, but not Bax-PGAM5S-Drp1. These data suggest that both arenobufagin and cisplatin inhibited the growth of CRC through apoptosis which depended on the formation of Bax-PGAM5L-Drp1 complex.

## DISCUSSION

Our experimental data indicated that PGAM5L, one of the two splice variants of the mitochondrial protein phosphatase PGAM5, is indispensable for the execution of intrinsic apoptosis. In suppressor of cytokine signaling 6 (SOCS6) induced intrinsic apoptosis, PGAM5 knockdown significantly alleviated SOCS6-mediated Bax activation and mitochondrial fragmentation [21]. However, it has been reported that staurosporine-induced apoptotic cell death was not affected by PGAM5 knockdown. Our results with shRNA targeting the 3′-untranslated region of PGAM5 are consistent with the results that the intrinsic apoptosis is partially dependent on PGAM5 [21]. These results are also in agreement with the recent report that trancated PGAM5 antagonized its binding with inhibitors of apoptotic protein (IAPs) and triggererd apoptosis through caspases activation. It has been proposed that PGAM5 released from the mitochondria is an early event in apoptosis and could steer the cell away from necrotic cell death by preventing mitochondrial binding of receptor-interacting protein (RIP) kinases [25].

PGAM5 contains a non-cleaved N-terminal mitochondrial targeting sequence that anchors PGAM5 to the cytosolic side of the outer mitochondrial membrane (OMM) [25, 26]. Upon induction of apoptosis, there is a significant release of PGAM5, especially PGAM5 (Δ24) into the cytosol that resembles the kinetics for Smac and cyto *C* [25]. However, it has recently been demonstrated that PGAM5 is predominantly localized to the inner mitochondrial membrane (IMM) with the C-terminal PGAM domain facing the intermembrane space [27]. It would also be possible that some membrane proteins escort the translocation of PGAM5 to the IMM [27]. Mitochondrial proteins destined for the inner membrane may have more hydrophobic membrane-spanning segments that function as stop-transfer sequences in the IMM or serve to insert the polypeptide into the IMM after it enters the matrix [28]. The possible ultra-micropositioning of PGAM5 variants and their roles in regulating cell death modes still need careful investigation [3, 25, 29].

It is the first time to show that Bax is required for PGAM5-Drp1 interaction in both arenobufagin and staurosporine induced intrinsic apoptosis. PGAM5 failed to interact with Drp1 in HCT116 Bax^−/−^ cells, which could be rescued by Bax transfection. Our results provided evidences of direct interaction between PGAM5 and Bax. The Bax was accumulated and oligomerized in mitochondria both in arenobufagin and staurosporine induced apoptosis [30]. The translocation of Bax formed pores on the OMM that were partially framed by a lipid monolayer [31]. iMAC2 significantly attenuated the interaction between PGAM5 and Drp1, further confirmed that Bax is necessary for the formation of PGAM5/Drp1 complex. Together our results suggest that Bax is not only an indispensable component of the lipid pore, but also a scaffold protein for the assembly of mitochondria attack complex consisted of PGAM5 and Drp1 [4, 32].

Drp1 is a protein of the mitochondrial fission machinery and has been reported to participate in apoptotic mitochondrial fragmentation. Drp1 is a key player in the regulation of mitochondrial dynamics. Oligomerization of Drp1 around mitochondria induces fission in a GTP-dependent manner [33]. Drp1 Ser637 dephosphorylation or Ser616 phosphorylation induce the mobilization of Drp1 to mitochondria and promote mitochondrial fragmentation [34, 35]. Bax has been reported to be co-localized with Drp1 at the mitochondrial constriction sites [36]. In another research, Drp1 stimulated tBid-induced Bax oligomerization and cyto *C* release [37]. Our results extended the fact that the co-lolalization is PGAM5 dependent because PGAM5 shRNA significantly attenuated the interaction between Drp1 and Bax. Arenobufagin induced the interaction between PGAM5 and Drp1 and further dephosphorylated Drp1 and induced mitochondrial fission.

Mitochondrial fission is an early apoptotic event occurring within the same time frame as activation of Bax and permeabilization of the mitochondrial outer membrane [38]. Bax and Drp1 function prior tocyto *C* release in the apoptotic cascade events, with Drp1 potentially participating in Bax activation [39]. Dominant-negative Drp1 inhibited mitochondrial fission and Bax accumulation on mitochondria which suggest that mitochondrial fission machinery acts upstream of Bax [40]. Then Bax recruitment and oligomerization at Drp1 foci may promote the stable association of Drp1 with membranes through inducing a shift in Drp1 cycling dynamics [39]. Constricting ability of Drp1 may also contribute to the formation of curvature within the outer membrane that is required for the efficient insertion of Bax [41]. Here we hypothesize a working model for the induction of intrinsic apoptosis: the mitochondrial PGAM5L acts as a beacon for the navigation of Drp1 to the mitochondria upon apoptosis induction, Bax swiftly translocates and inserts to the mitochondrial outer membrane and acts as a scaffold for the interaction of PGAM5 and Drp1. Subsequently, MOMP and mitochondria fission are induced [42], cyto *C* and AIF are released into the cytosol. The former activates the caspase cascade and the later translocates to the nuclear to induce intrinsic apoptosis.

The orthotopical and heterotropic models showed the efficacy of arenobufagin in preventing the growth and metastasis of CRC, suggesting its therapeutic potential as an anti-CRC agent with less toxicity. The formation of Bax-PGAM5L-Drp1 complex can be observed *in vitro* in cells treated with arenobufagin and staurosprine. The complex can also be induced by arenobufagin and cisplatin *in vivo* in Bax^+/+^ tumors. These data provide the first evidences that PGAM5L controls the Bax activation and Drp1 dephosphorylation and induces mitochondria fission. Bax-PGAM5L-Drp1 complex is a potential oncotarget and is required for inducing intrinsic apoptosis.

## MATERIALS AND METHODS

### Cell culture and transfection

Human Colon cell lines (SW480, HCT-116 and LS174T) and HCT-116 Bax^−/−^ were obtained from American Type Culture Collection (ATCC, Rockville, MD). SW480 and HCT-116 were incubated in RPMI-1640 (Invitrogen), LS174T incubated in DMEM (Invitrogen), and HCT-116 Bax^−/−^ incubated in McCoy's 5A (Gibco). All cell lines were supplemented with 10% (v/v) fetal bovine serum (Invitrogen) and 1% (v/v) penicillin–streptomycin (Invitrogen) at 37°C in a humidified atmosphere of 5% CO_2_. siRNA transient transfections were performed using Lipofectamine 2000 (Invitrogen, Carlsbad, CA, USA) according to the instructions of the manufacturers.

### Lentiviral preparation, viral infection, and stable cell generation

The pLKO.1-shRNA plasmids encoding shRNAs with sequences targeting human PGAM5L and PGAM5S were purchased from the GenePharma Facility (Suzhoui, China). The shRNA sequence of shRNA-PGAM5L contained 5′-TATTGGGCTGTCACTAGCGTG-3′, and shRNA-PGAM5S contained 5′,-TTGGCCAACCCTTCTGACT-3′. The shRNA targeting firefly luciferase 5′,-TTCTCCGAA CGTGTCACGTTTC-3′ was incorporated as a control. Viruses were collected from the medium 60 h after transfection. For KD experiments, cells were infected with the collected viruses over 24 h in the presence of polybrene, followed by selection in medium containing puromycin (0.5 mg/ml) for 7–9 days as described previously.

### Bax oligomerization

To detect the Bax oligomerization, cells were washed with phosphate-buffered saline (PBS) and treated with 10 mM of the crosslinking agent disuccinimidyl suberate (DSS) (Sigma) in dimethylsulfoxide (DMSO) at room temperature for 30 min. After quenching the crosslinker by the addition of 1M Tris-HCl (pH 7.4) to a final concentration of 20 mM for 10 mins, cells were lysed for further analysis.

### Immunofluorescence

Cells were stained with MitoTracker. The cells were incubated with antibody at 4°C overnight, incubated with Cy3-labeled goat anti-rabbit or anti-mouse IgG antibody (1:500) in darkness for 60 mins at room temperature, and then counterstained with 4′6-diamidino-2-phenylindole (DAPI), and examined under confocal microscope (Nikon, Japan) with excitation and emission wavelengths of 550 and 570 nm, respectively with a 100 × 1.40 NA oil immersion objective. For mitochondrial staining, 100 nM MitoTracker Green (Molecular Probes) was added to cultures 30 mins before fixation.

### Mitochondrial morphology analysis (Micro-P)

Cells were stained with MitoTracker and cell images were taken with confocal microscope. Cells were then analyzed by Micro-P software. Micro-P software measured individual mitochondrion according to the area, axial, and length/width, and sorted mitochondria into five types.

### Immunoprecipitation

Cell extract was mixed with anti-PGAM5, anti-Bax, and anti-Drp1 antibody at 4°C overnight. Agarose beads were added at a ratio of 1 mg of extract per 30 ml of agarose at 4°C for 3 h. The beads were then pelleted at 2,500 × g for 3 min and washed with lysis buffer five times. The beads were subjected to elution with 5 vol of 0.5 mg/ml peptide for 4 h or directly boiled in loading buffer.

### Orthotopic CRC model and xenograft CRC model

All animal research procedures were conformed to the guidelines for The Care and Use of Laboratory Animals published by the National Institutes of Health and were approved by The Laboratory Animals Care and Use Committee of Southern Medical University. SW480-eGFP (5 × 10^6^) cells were injected subcutaneously to nude mice. Ten days later. Tumor tissue was divided into small pieces, approximately 1 mm^3^. Nude mice were anesthetized, and surgical orthotopic implantation (SOI) of tumor in colon was performed. Animals were kept in a sterile environment. To investigate tumor metastasis, various organs were collected from the tumor-bearing mice.

HCT-116 and HCT-116 Bax^−/−^ cells (1 × 10^7^) were inoculated subcutaneously on the groin of nude mice and allowed to grow for ∼7 days to reach a tumor volume of ∼50 mm^3^. Their body weights and tumor volumes were measured every 5 days throughout the treatment period. The mice were euthanized at the end of the experiments. Tumor xenografts were removed and weighed.

### PET-CT

All mice were fasted for 17 hours. Then, under 2% isoflurane anesthesia, the mice were administered 18F-FDG intravenously. Following the injections, mice were left awake for 60 minutes and subsequently placed in the supine position in a mouse holder and anesthetized with 2% isoflurane for upper-body PET imaging. The mouse holder was placed on the PET/CT (Inveon Micro-PET/CT, SIMENS) bed and all animals had a CT scan after the PET scan for attenuation correction and anatomical delineation of PET images.

### Statistical analyses

The results were expressed as the means ± S.E.M. The *P*-values were two-tailed and performed using Student's *t*-test. Statistical significance was specified as *P* < 0.05.

## SUPPLEMENTARY EXPERIMENTAL PROCEDURES FIGURES



## References

[1] Siegel R, Ma J, Zou Z, Jemal A (2014). Cancer statistics, 2014. CA Cancer J Clin.

[2] Fernald K, Kurokawa M (2013). Evading apoptosis in cancer. Trends Cell Biol.

[3] Wang Z, Jiang H, Chen S, Du F, Wang X (2012). The mitochondrial phosphatase PGAM5 functions at the convergence point of multiple necrotic death pathways. Cell.

[4] Chan FK, Baehrecke EH (2012). RIP3 finds partners in crime. Cell.

[5] Wolff S, Erster S, Palacios G, Moll UM (2008). p53's mitochondrial translocation and MOMP action is independent of Puma and Bax and severely disrupts mitochondrial membrane integrity. Cell Res.

[6] Zhang DM, Liu JS, Deng LJ, Chen MF, Yiu A, Cao HH, Tian HY, Fung KP, Kurihara H, Pan JX, Ye WC (2013). Arenobufagin, a natural bufadienolide from toad venom, induces apoptosis and autophagy in human hepatocellular carcinoma cells through inhibition of PI3K/Akt/mTOR pathway. Carcinogenesis.

[7] Mao YW, Liu JP, Xiang H, Li DW (2004). Human alphaA- and alphaB-crystallins bind to Bax and Bcl-X(S) to sequester their translocation during staurosporine-induced apoptosis. Cell Death Differ.

[8] Zhang XD, Gillespie SK, Hersey P (2004). Staurosporine induces apoptosis of melanoma by both caspase-dependent and -independent apoptotic pathways. Mol Cancer Ther.

[9] Kroemer G, Galluzzi L, Vandenabeele P, Abrams J, Alnemri ES, Baehrecke EH, Blagosklonny MV, El-Deiry WS, Golstein P, Green DR, Hengartner M, Knight RA, Kumar S, Lipton SA, Malorni W, Nunez G (2009). Classification of cell death: recommendations of the Nomenclature Committee on Cell Death 2009. Cell Death Differ.

[10] He S, Wang L, Miao L, Wang T, Du F, Zhao L, Wang X (2009). Receptor interacting protein kinase-3 determines cellular necrotic response to TNF-alpha. Cell.

[11] Peixoto PM, Ryu SY, Bombrun A, Antonsson B, Kinnally KW (2009). MAC inhibitors suppress mitochondrial apoptosis. Biochem J.

[12] Kroemer G, Reed JC (2000). Mitochondrial control of cell death. Nat Med.

[13] Arnoult D, Gaume B, Karbowski M, Sharpe JC, Cecconi F, Youle RJ (2003). Mitochondrial release of AIF and EndoG requires caspase activation downstream of Bax/Bak-mediated permeabilization. EMBO J.

[14] Galluzzi L, Vitale I, Abrams JM, Alnemri ES, Baehrecke EH, Blagosklonny MV, Dawson TM, Dawson VL, El-Deiry WS, Fulda S, Gottlieb E, Green DR, Hengartner MO, Kepp O, Knight RA, Kumar S (2012). Molecular definitions of cell death subroutines: recommendations of the Nomenclature Committee on Cell Death 2012. Cell Death Differ.

[15] Cassidy-Stone A, Chipuk JE, Ingerman E, Song C, Yoo C, Kuwana T, Kurth MJ, Shaw JT, Hinshaw JE, Green DR, Nunnari J (2008). Chemical inhibition of the mitochondrial division dynamin reveals its role in Bax/Bak-dependent mitochondrial outer membrane permeabilization. Dev Cell.

[16] Tailor D, Hahm ER, Kale RK, Singh SV, Singh RP (2014). Sodium butyrate induces DRP1-mediated mitochondrial fusion and apoptosis in human colorectal cancer cells. Mitochondrion.

[17] Qian W, Choi S, Gibson GA, Watkins SC, Bakkenist CJ, Van Houten B (2012). Mitochondrial hyperfusion induced by loss of the fission protein Drp1 causes ATM-dependent G2/M arrest and aneuploidy through DNA replication stress. J Cell Sci.

[18] Qian W, Wang J, Van Houten B (2013). The role of dynamin-related protein 1 in cancer growth: a promising therapeutic target?. Expert Opin Ther Targets.

[19] Estaquier J, Arnoult D (2007). Inhibiting Drp1-mediated mitochondrial fission selectively prevents the release of cytochrome c during apoptosis. Cell Death Differ.

[20] Tanaka A, Youle RJ (2008). A chemical inhibitor of DRP1 uncouples mitochondrial fission and apoptosis. Mol Cell.

[21] Lin HY, Lai RH, Lin ST, Lin RC, Wang MJ, Lin CC, Lee HC, Wang FF, Chen JY (2013). Suppressor of cytokine signaling 6 (SOCS6) promotes mitochondrial fission via regulating DRP1 translocation. Cell Death Differ.

[22] Brooks C, Cho SG, Wang CY, Yang T, Dong Z (2011). Fragmented mitochondria are sensitized to Bax insertion and activation during apoptosis. Am J Physiol Cell Physiol.

[23] Chunhua L, Donglan L, Xiuqiong F, Lihua Z, Qin F, Yawei L, Liang Z, Ge W, Linlin J, Ping Z, Kun L, Xuegang S (2013). Apigenin up-regulates transgelin and inhibits invasion and migration of colorectal cancer through decreased phosphorylation of AKT. J Nutr Biochem.

[24] Ruegg UT, Burgess GM (1989). Staurosporine, K-252 and UCN-01: potent but nonspecific inhibitors of protein kinases. Trends Pharmacol Sci.

[25] Zhuang M, Guan S, Wang H, Burlingame AL, Wells JA (2013). Substrates of IAP ubiquitin ligases identified with a designed orthogonal E3 ligase, the NEDDylator. MOL Cell.

[26] Lo SC, Hannink M (2008). PGAM5 tethers a ternary complex containing Keap1 and Nrf2 to mitochondria. Exp Cell Res.

[27] Sekine S, Kanamaru Y, Koike M, Nishihara A, Okada M, Kinoshita H, Kamiyama M, Maruyama J, Uchiyama Y, Ishihara N, Takeda K, Ichijo H (2012). Rhomboid protease PARL mediates the mitochondrial membrane potential loss-induced cleavage of PGAM5. J Biol Chem.

[28] Herrmann JM, Longen S, Weckbecker D, Depuydt M (2012). Biogenesis of mitochondrial proteins. Adv Exp Med Biol.

[29] Zhao J, Jitkaew S, Cai Z, Choksi S, Li Q, Luo J, Liu ZG (2012). Mixed lineage kinase domain-like is a key receptor interacting protein 3 downstream component of TNF-induced necrosis. Proc Natl Acad Sci U S A.

[30] Zhang XD, Gillespie SK, Hersey P (2004). Staurosporine induces apoptosis of melanoma by both caspase-dependent and -independent apoptotic pathways. Mol Cancer Ther.

[31] Qian S, Wang W, Yang L, Huang HW (2008). Structure of transmembrane pore induced by Bax-derived peptide: evidence for lipidic pores. Proc Natl Acad Sci U S A.

[32] Zhou Z, Han V, Han J (2012). New components of the necroptotic pathway. Protein Cell.

[33] Smirnova E, Griparic L, Shurland DL, van der Bliek AM (2001). Dynamin-related protein Drp1 is required for mitochondrial division in mammalian cells. Mol Biol Cell.

[34] Cereghetti GM, Stangherlin A, Martins DBO, Chang CR, Blackstone C, Bernardi P, Scorrano L (2008). Dephosphorylation by calcineurin regulates translocation of Drp1 to mitochondria. Proc Natl Acad Sci U S A.

[35] Taguchi N, Ishihara N, Jofuku A, Oka T, Mihara K (2007). Mitotic phosphorylation of dynamin-related GTPase Drp1 participates in mitochondrial fission. J BIOL CHEM.

[36] Karbowski M, Lee YJ, Gaume B, Jeong SY, Frank S, Nechushtan A, Santel A, Fuller M, Smith CL, Youle RJ (2002). Spatial and temporal association of Bax with mitochondrial fission sites, Drp1, and Mfn2 during apoptosis. J Cell Biol.

[37] Montessuit S, Somasekharan SP, Terrones O, Lucken-Ardjomande S, Herzig S, Schwarzenbacher R, Manstein DJ, Bossy-Wetzel E, Basanez G, Meda P, Martinou JC (2010). Membrane remodeling induced by the dynamin-related protein Drp1 stimulates Bax oligomerization. Cell.

[38] Martinou JC, Youle RJ (2006). Which came first, the cytochrome c release or the mitochondrial fission?. Cell Death Differ.

[39] Wasiak S, Zunino R, McBride HM (2007). Bax/Bak promote sumoylation of DRP1 and its stable association with mitochondria during apoptotic cell death. J Cell Biol.

[40] Yuan H, Gerencser AA, Liot G, Lipton SA, Ellisman M, Perkins GA, Bossy-Wetzel E (2007). Mitochondrial fission is an upstream and required event for bax foci formation in response to nitric oxide in cortical neurons. Cell Death Differ.

[41] Basanez G, Sharpe JC, Galanis J, Brandt TB, Hardwick JM, Zimmerberg J (2002). Bax-type apoptotic proteins porate pure lipid bilayers through a mechanism sensitive to intrinsic monolayer curvature. J Biol Chem.

[42] Landes T, Martinou JC (2011). Mitochondrial outer membrane permeabilization during apoptosis: the role of mitochondrial fission. Biochim Biophys Acta.

