# Supplementary material for: Bax-PGAM5L-Drp1 complex is required for intrinsic apoptosis execution
Source: Oncotarget. 2015 Aug 31;6(30):30017–34. doi: 10.18632/oncotarget.5013 (PMC4745779; doi:10.18632/oncotarget.5013)
Supplement: Supplementary file 1 [file oncotarget-06-30017-s001.pdf]

SUPPLEMENTARY EXPERIMENTAL PROCEDURES

Reagents

PGAM5L, PGAM5S and Drp1 siRNA kits were purchased from Genepharma (Shanghai, China). Thiazolyl blue tetrazolium bromide (MTT), Mdivi-1 and Staurosporine(STS) were purchased from Sigma-Aldrich (St. Louis, MO, USA). Hoechst33342 and iMAC2 were purchased from Merck Millipore (Germany). TUNEL Kit were purchased from Roche (USA). Z-VAD-fmk was from Bachem. PE-Annexin V Apoptpsis Detection Kit were purchased from BD (USA). The following antibodies were used: anti-Caspase-3 (Cell Signaling, 9662), anti-Caspase-9 (Cell Signaling, 9502), anti-Caspase-7 (Cell Signaling, 9492), anti-PARP (Cell Signaling Tech, 9542), anti-Bax (Cell Signaling, 2772),anti-Bcl-2 (Cell Signaling, 2870), anti-Cytochrome c (Cell Signaling, 4272), anti-AIF (Cell Signaling, 4642), Anti-PGAM5(Abcam, ab126534, corresponding to amino acids 61–146 of human PGAM5), anti-Drp1 (Abcam, ab56788), anti-phospho-Drp1 (ser616) (Cell Signaling, 3455), and anti-phospho-Drp1 (ser637) (GeneTex, GTX50911).

Detection of apoptosis and mitochondrial membrane potential

Apoptosis was detected by Hoechst33342 fluorescent staining and PE-Annexin V/7-Amino-Actinomycin(7-AAD) apoptosis detection kit (BD Pharmingen, USA) according to the manufacturer's protocol. The changes in mitochondrial membrane potential were measured by staining cells with 1.5 mM JC-1 (Molecular Probes) at 37°C for 30 min before flow cytometric analysis.

Western blot analysis

Preparation of total protein lysates and western blot analysis were performed as described previously. The cytosol, nucleus and mitochondrial fractions of cells were isolated and immunoblotted with anti-Bax, anti-Bcl-2, anti-Cytochrome c, and anti-AIF as described previously.

Orthotopic CRC model

SW480-eGFP (5×10^6) cells were injected subcutaneously to BALB/c-nu. Ten days later, the tumors were removed and washed twice with RPMI 1640 medium containing antibiotics. Tumor tissue was divided into small pieces, approximately 1 mm^3. Nude mice were anesthetized, and colon surgical orthotopic implantation (SOI) of tumor was performed [1]. Mice were randomly divided into three groups with ten mice in each group. Control group was administered a daily intraperitoneal dose of 0.1% DMSO in PBS. The other two groups were administered daily with a low dosage of arenobufagin (3 mg/kg/day intraperitoneal) or high dosage of arenobufagin (6 mg/kg/day intraperitoneal).

Xenograft CRC model

Mice inoculated HCT-116 and HCT-116 $Bax^{-/-}$ cells were randomly divided into six groups with twelve mice in each group [2]. Two of the groups cells was administered a daily dose of 0.1% DMSO as the vehicle control. The other two groups were administered daily with Arenobufagin (3 mg/kg/day intraperitoneal) or Cisplatin (10 mg/kg/3 days intraperitoneal).

Immunohistochemical and TUNEL

Paraffin-embedded colon sections were dewaxed, rehydrated, and pre-treated with hydrogen peroxidase in PBS buffer. Heat-induced antigen retrieval was performed. Sections were incubated with anti-PGAM5, anti-Drp1, and anti-Bax. After incubation with HRP-conjugated secondary antibody and tyramide amplification followed by streptavidin-HRP, positive signals were visualized by DAB kit. Section were examined at a magnification of 400× and analyzed using NIS-Elements. The quantification of apoptosis of Immunohistochemical detection was based on TUNEL technology (labeling of DNA strand breaks), analysised by light microscopy.

REFERENCES

1. Chunhua L, Donglan L, Xiuqiong F, Lihua Z, Qin F, Yawei L, Liang Z, Ge W, Linlin J, Ping Z, Kun L, Xuegang S. Apigenin up-regulates transgelin and inhibits invasion and migration of colorectal cancer through decreased phosphorylation of AKT. J Nutr Biochem. 2013; 24:1766–1775.
2. Sakamori R, Yu S, Zhang X, Hoffman A, Sun J, Das S, Vedula P, Li G, Fu J, Walker F, Yang CS, Yi Z, Hsu W, Yu DH, Shen L, Rodriguez AJ, et al. CDC42 Inhibition Suppresses Progression of Incipient Intestinal Tumors. Cancer Res. 2014; 74:5480–5492.

SUPPLEMENTARY FIGURES AND VIDEOS

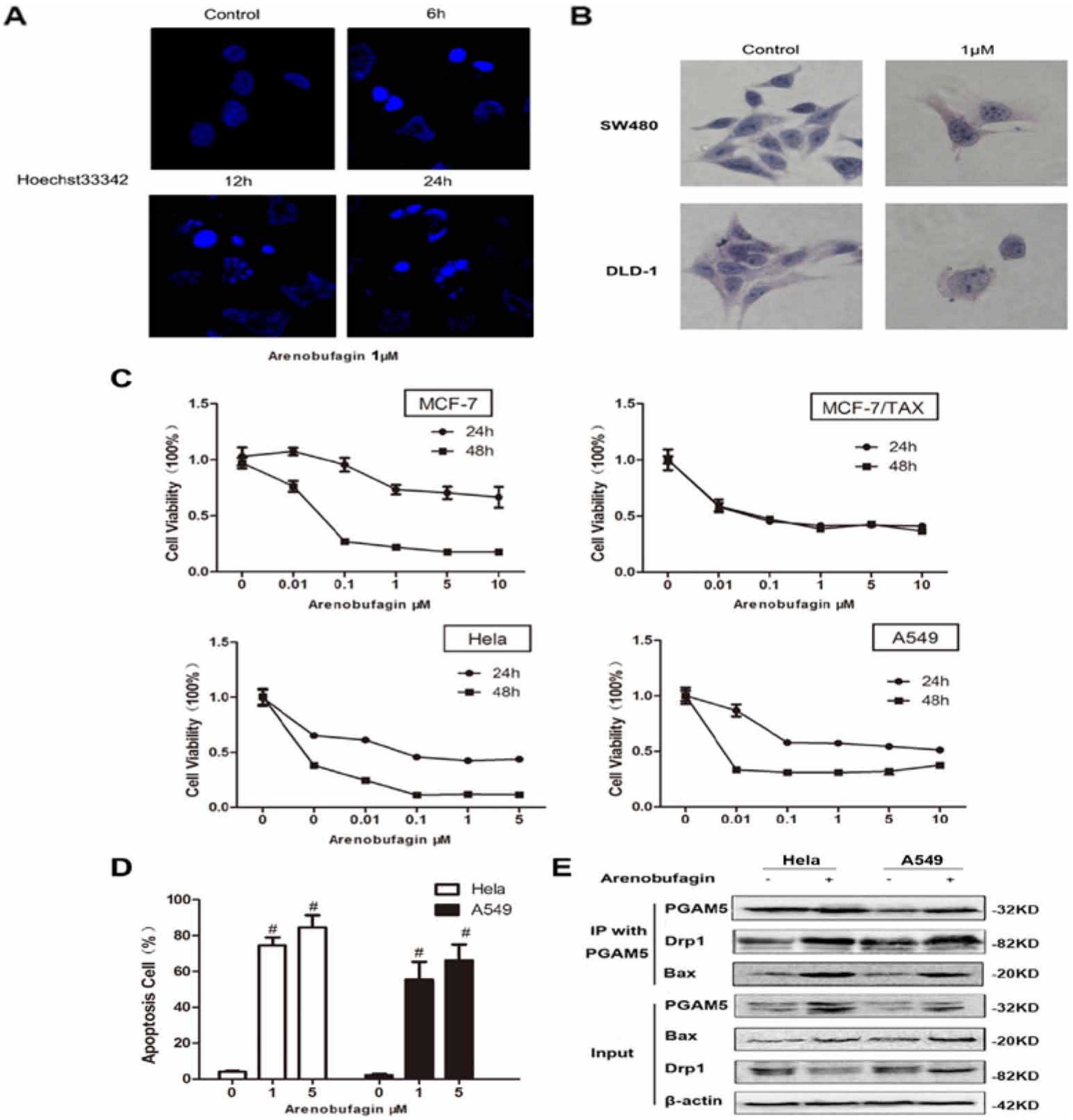

Supplementary Figure S1, related to Figure 1: A. SW480 cells were incubated with arenobufagin for indicated time and stained with Hoechst33342 (5 μg/ml) for 15 min at room temperature. Fluorescence was analyzed by confocal fluorescence microscopy. 1000× for all, scale bar = 10 μm. **B.** Hematoxylin-Eosin staining of SW480 and DLD-1 cells. 400× for all, scale bar = 10 μm. **C.** Effects of Arenobufagin on proliferation of Hela, A549, MCF-7 and MCF-7/TAX. Cells were exposed to either vehicle or 0.01–10 μM Arenobufagin and incubated for 24 and 48 h. Cell viability was measured by MTT methods. **D.** The proportion of annexin V-positive cells was analyzed by flow cytometry in Hela and A549 cells (n = 6 per group). **E.** Hela and A549 cells were treated with 1 μM arenobufagin for 24 h. IP was performed with an anti-PGAM5 antibody. Co-IP Drp1 and Bax were detected by western blotting (n = 3).

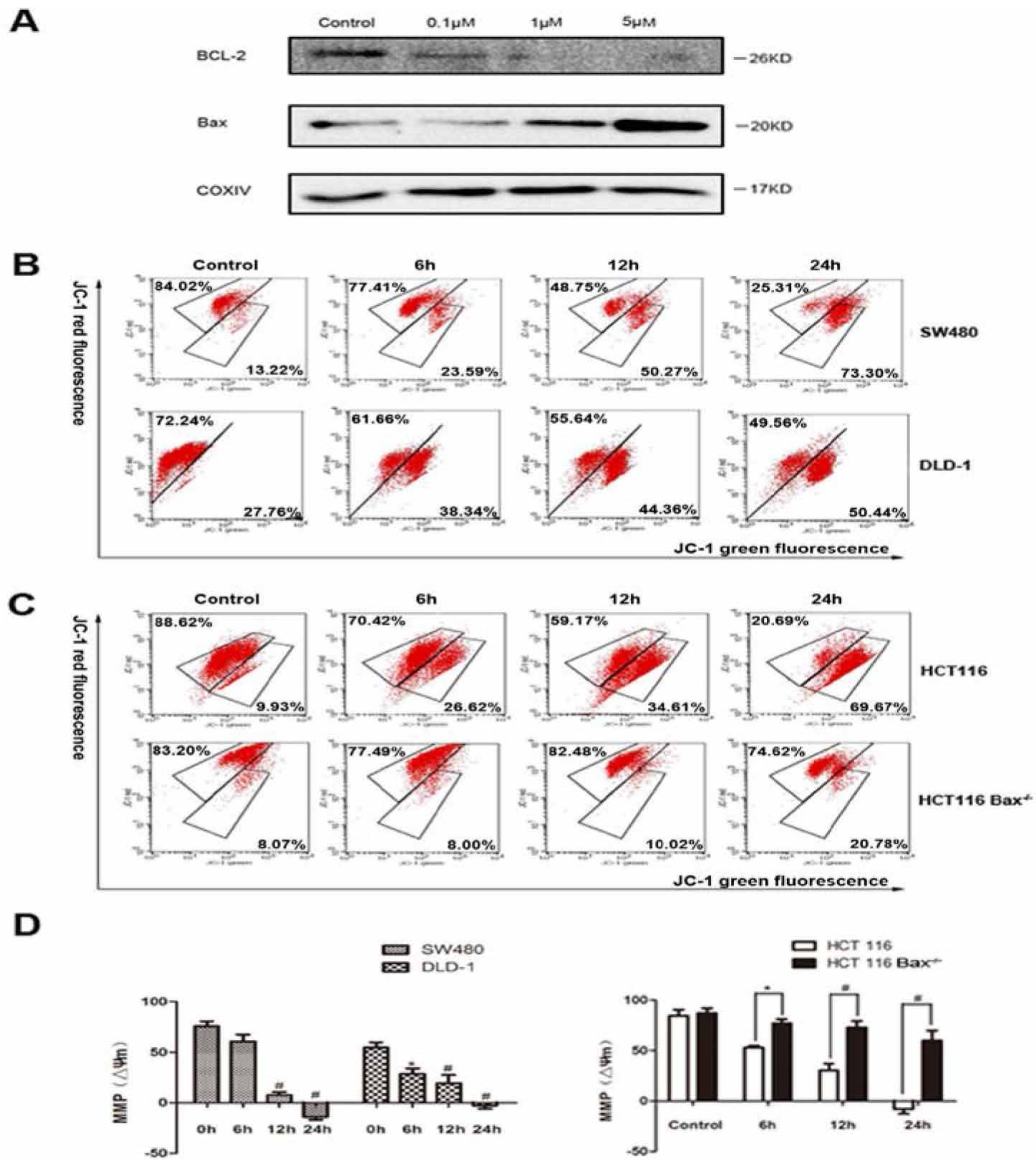

Supplementary Figure S2, related to Figure 2: A. Effects of arenobufagin on translocation of Bax and Bcl-2 in the mitochondria. Level of Bax and Bcl-2 in mitochondrial fractions from sw480 treated with arenobufagin at the indicated concentrations for 24 h were evaluated by western blot analysis. **B–C.** Arenobufagin depolarizes mitochondrial membran potential in hepatoma carcinoma cells. SW480, DLD-1, HCT116 WT and HCT116 $Bax^{-/-}$ cells were treated arenobufagin for different time, then mitochondrial membrane potential was evaluated with flow cytometry after stained with fluorescent dye, JC-1. Distributions of red (JC-1 aggregates) versus green (JC-1 monomers) fluorescence in cells were shown. **D.** HCT116 WT and HCT116 $Bax^{-/-}$ mitochondrial membrane potential were quantitatively analyzed. $^{*}P < 0.05$, $^{\#}P < 0.01$, one-way ANOVA, post hoc comparisons, Tukey,s test. Columns, means; error bars, SEs.

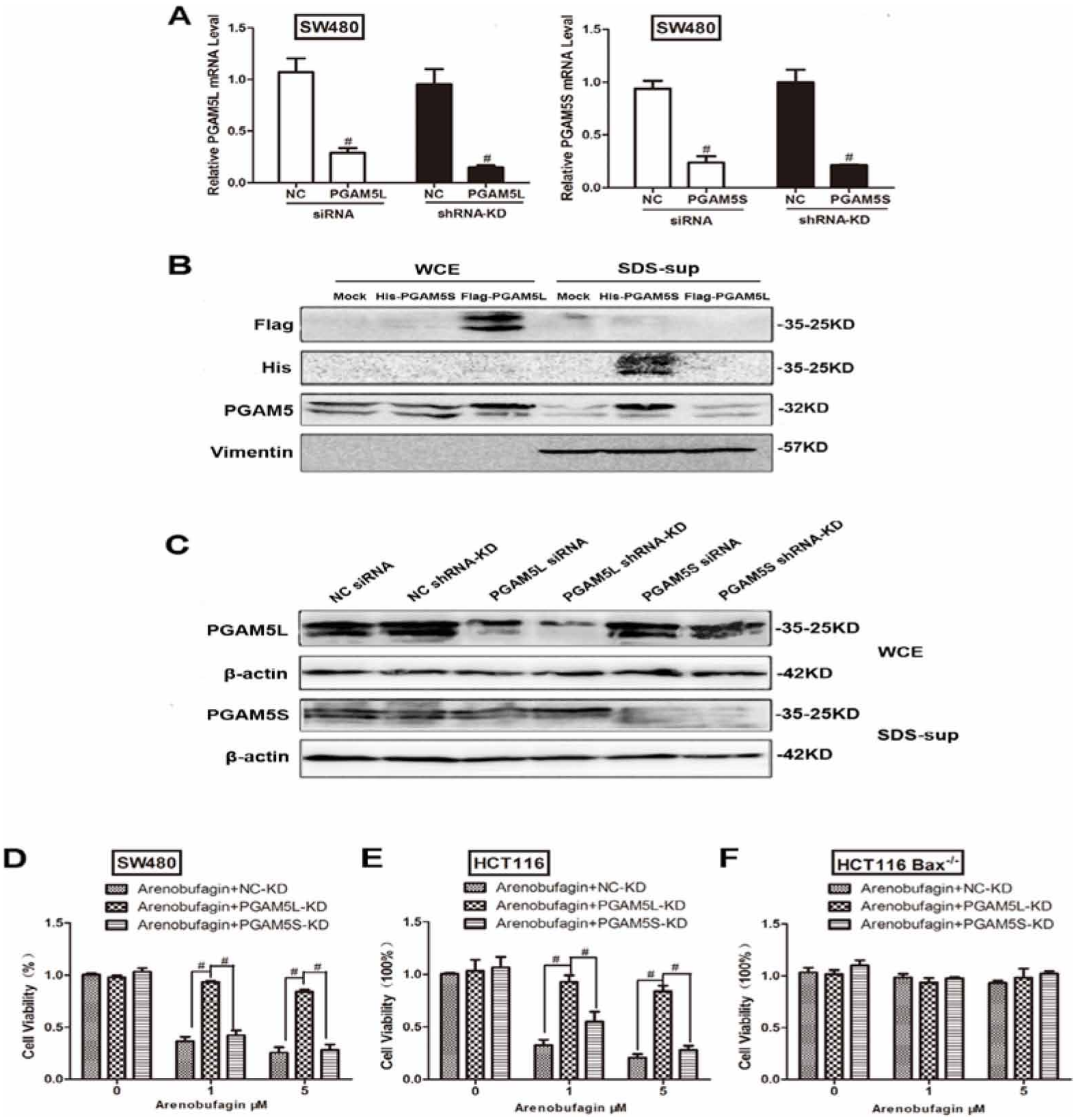

Supplementary Figure S3, related to Figure 3: A. Gene expression analysis of PGAM5L and PGAM5S of colon cells by RT-qPCR. The relative quantification value, fold difference, is expressed as $2^{-\Delta\Delta Ct}$. vs. NC, *: $P < 0.05$, $^{\#}$: $P < 0.01$, n.s.: not significant. **B.** PGAM5S is localized in an SDS-soluble domain. SW480 cells were transfected with a plasmid expressing His-PGAM5S or Flag-PGAM5L. After isolating whole-cell extracts (WCEs), the pellet was further extracted with 1% SDS. Western blotting was performed with anti-His, anti-Flag, anti-PGAM5, and anti-vimentin antibodies. **C.** Comparing the knockdown efficiency of isoform specific siRNA or shRNA. For PGAM5L, SW480 cells were transfected with the siRNA or shRNA, and WCE was used for western blottingWestern blotting was performed with anti-PGAM5 antibody. For PGAM5S, the siRNA or shRNA was transfected into cells. After 72 hr, the WCE was discarded, and the cell pellet was directly boiled in 1% SDS loading buffer. Western blotting was performed with anti-PGAM5 antibody. SW480, HCT116 WT and HCT116 Bax$^{-/-}$ cells were treated with arenobufagin in the absence or presence of PGAM5L-KD or PGAM5S-KD **D–F.** Cell viability was measured by MTT assay (n = 6 per group).

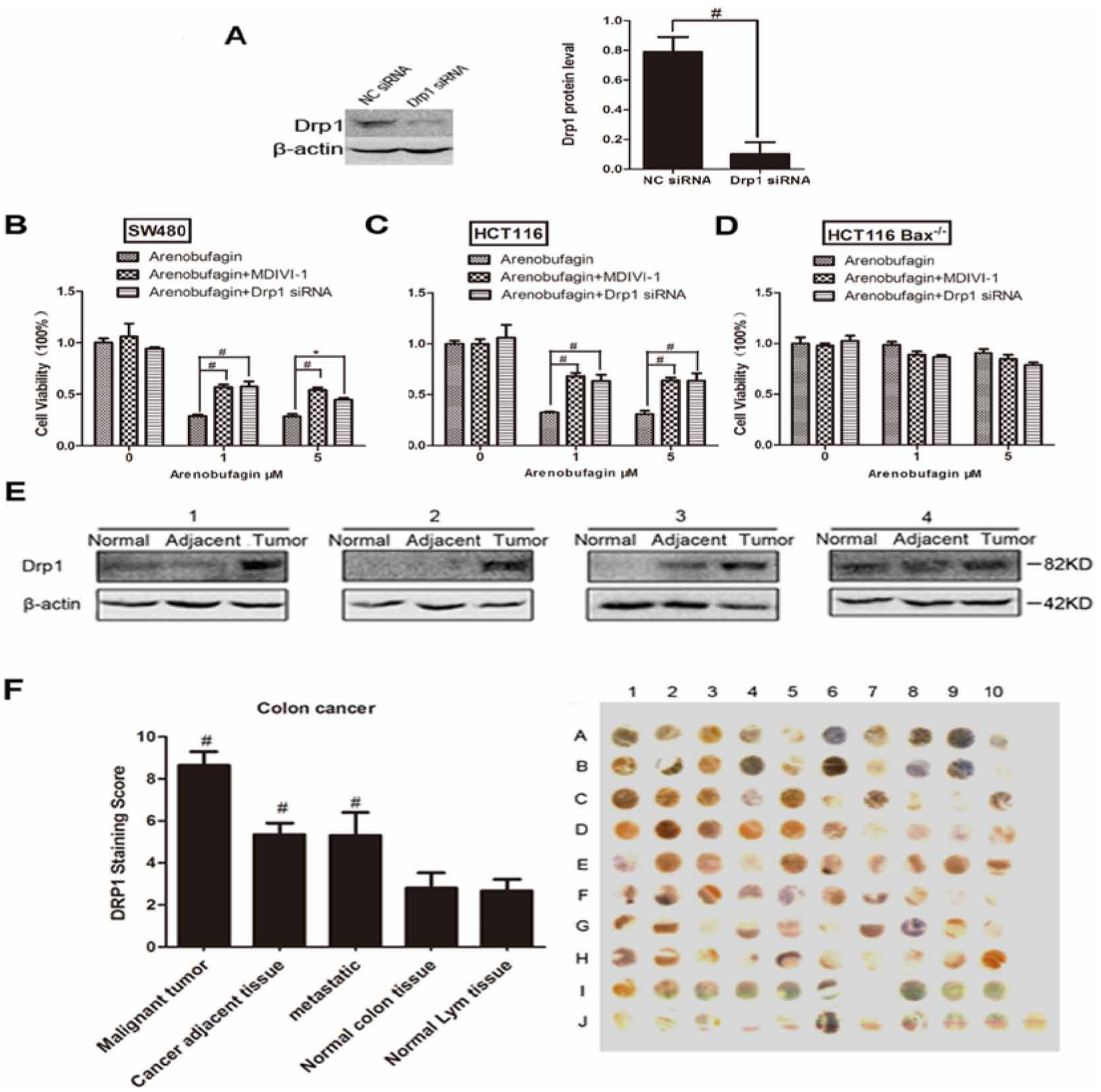

Supplementary Figure S4, related to Figure 4: A. Cells were transfected with Drp1 siRNA(100 nM). Immunoblot analysis of Drp1 were determined after 48 h. SW480, HCT116 WT and HCT116 Bax$^{-/-}$ cells were treated with arenobufagin in the absence or presence of Drp1 siRNA (100 nM), or Mdivi-1 (50 μM) **B–D.** Cell viability was measured by MTT assay ($n = 6$ per group). **E.** Western blot analysis for Drp1 in 4 paired specimens of primary colorectal tumor, adjacent, and normal mucosa. The study protocol was reviewed and approved by the institutiona review board of Guangdong General Hospital. **F.** Immunohistochemical staining was performed using the Histostain-Plus Kit (DAB, Invitrogen) and assessed blindly by two independent investigators.(A1~D6: Malignant tumor, D7~I1: Adjacent tissue 1.5 cm away from tumor, I2~I10: Normal lymph node tissue, J1~J10: Normal colon tissue.) *$P <$ 0.05, $^{\#}P < 0.01$, one-way ANOVA, post hoc comparisons, Tukey,s test. Columns, means; error bars, SEs.

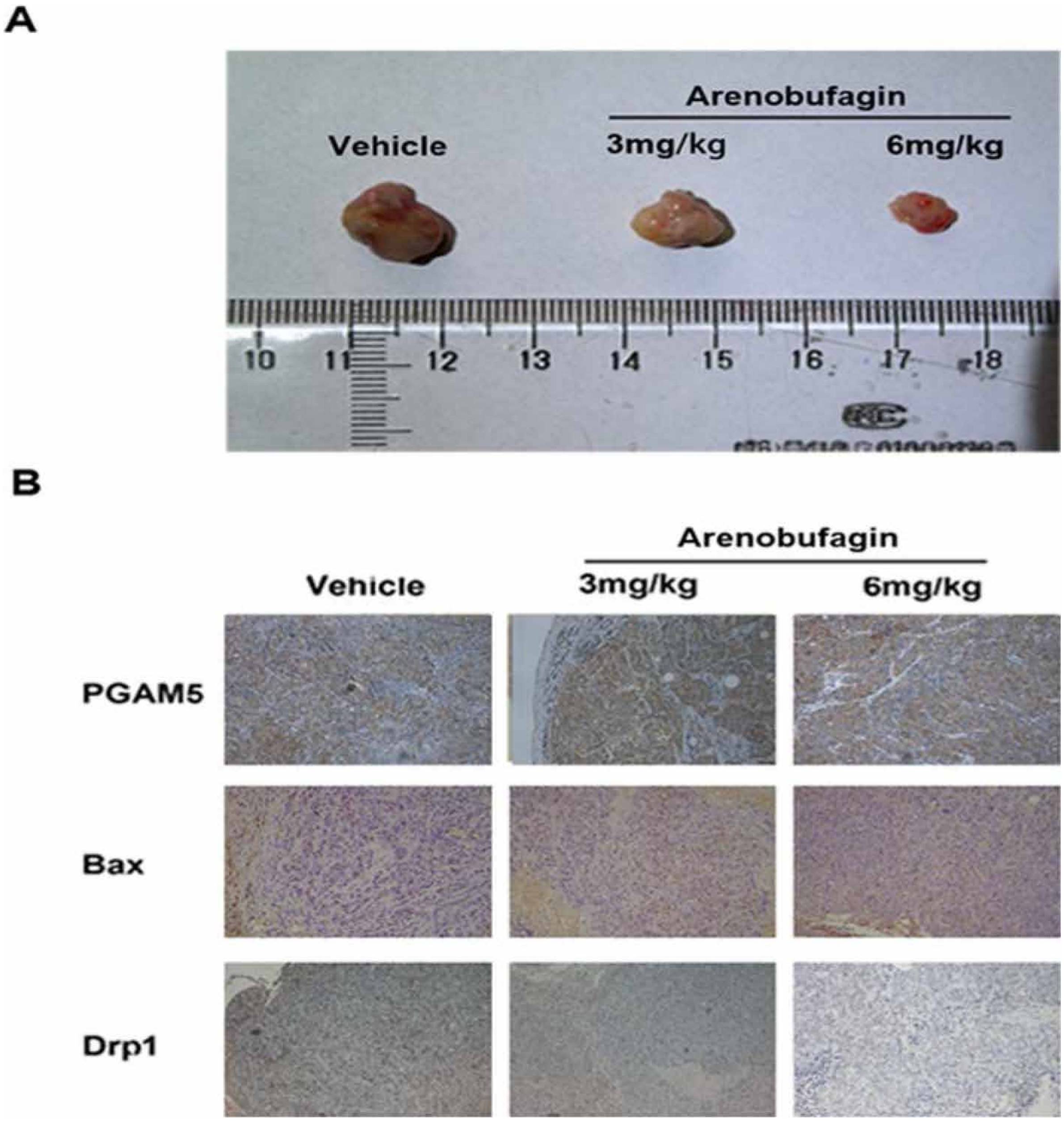

Supplementary Figure S5, related to Figure 5: A. Microscopic view of colon tumor tissue in mice. **B.** Immunohistochemical staining of PGAM5, Drp1 and Bax expression in tumor tissues or normal colonic tissues. 200× for all, scale bar = 100 µm.

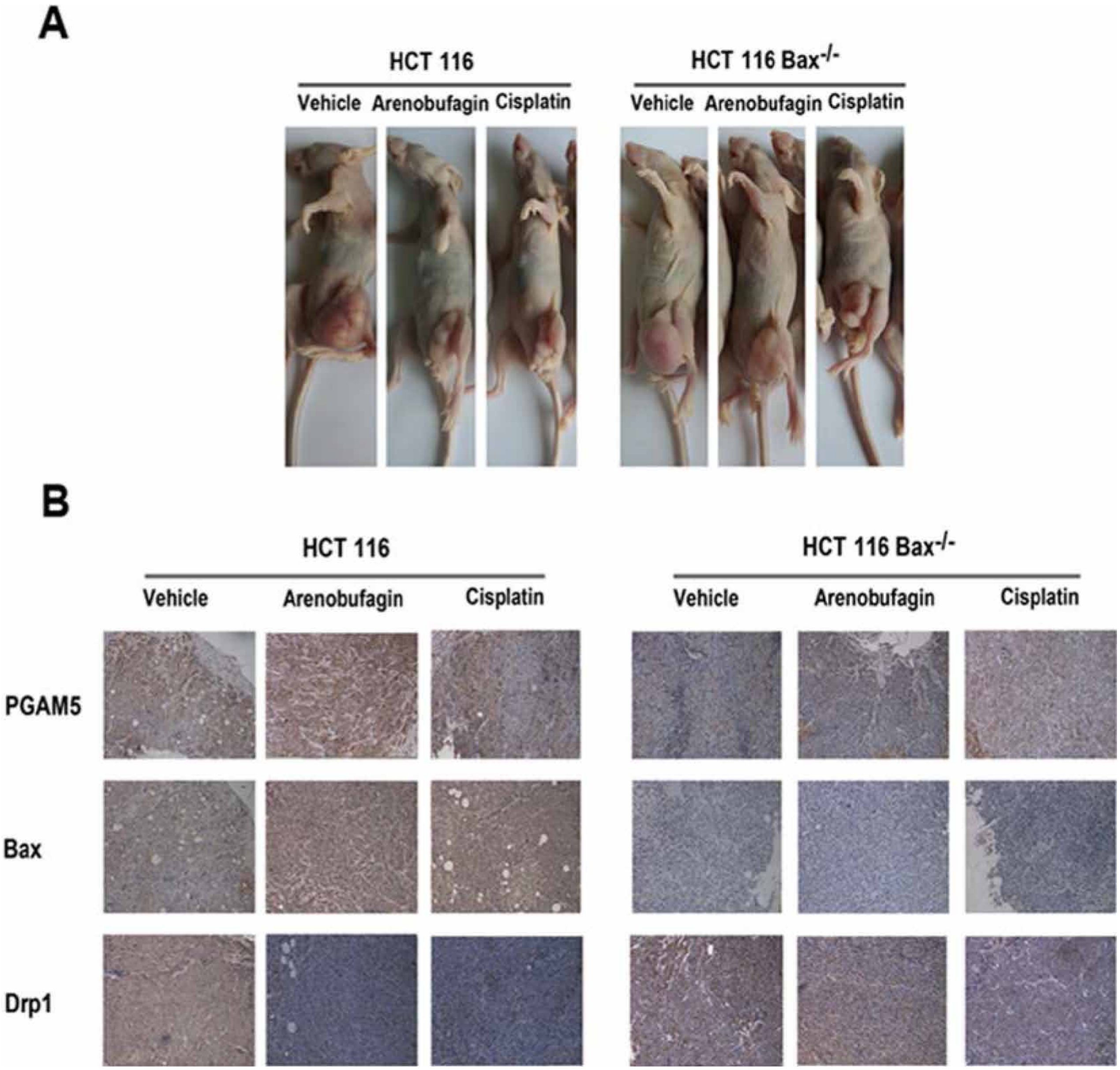

Supplementary Figure S6, related to Figure 6: A. View of tumors *in vivo*. **B.** Immunohistochemical staining of PGAM5, Drp1 and Bax expression in tumor tissues or normal colonic tissues. 200× for all, scale bar = 100 µm.

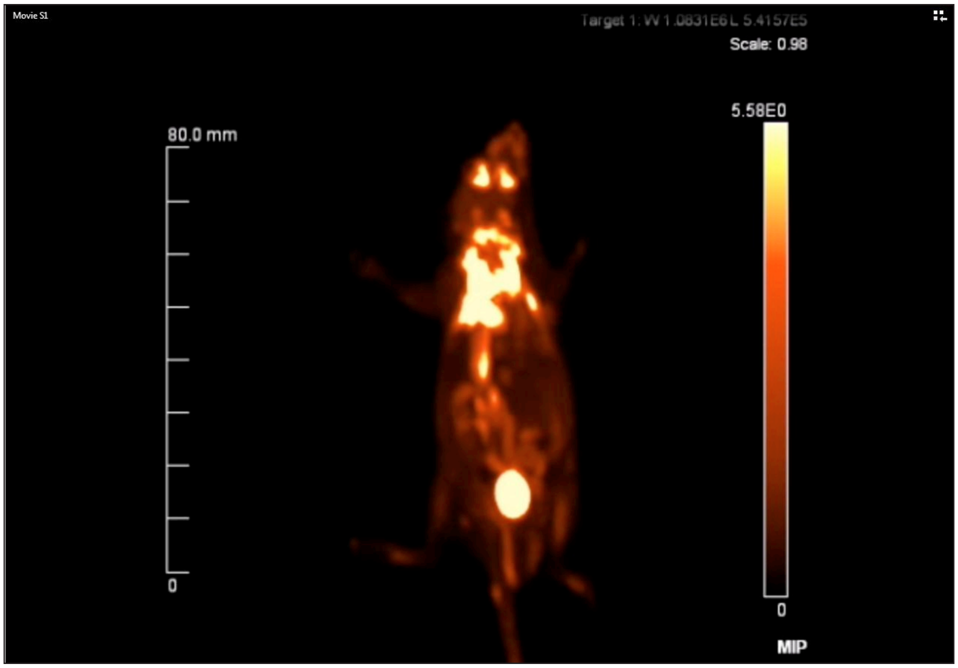

Supplementary Video S1: PET-CT Video of mice in blank group, Related to Figure 8.

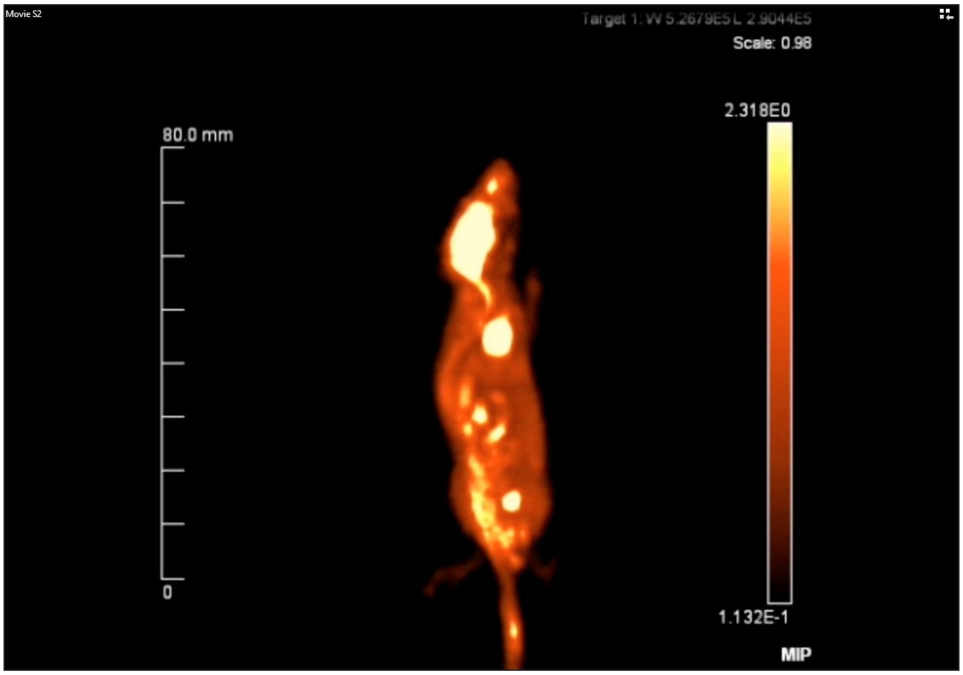

Supplementary Video S2: PET-CT Video of mice in vehicle group, Related to Figure 8.

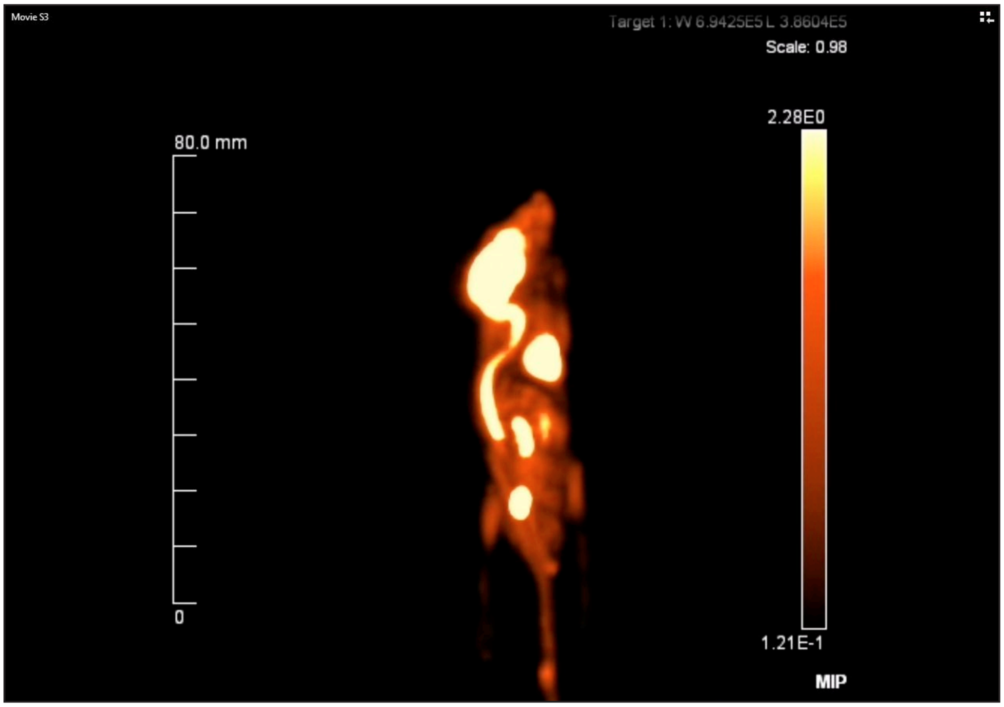

Supplementary Video S3: PET-CT Video of mice in 3 mg/kg arenobufagin group, Related to Figure 8.

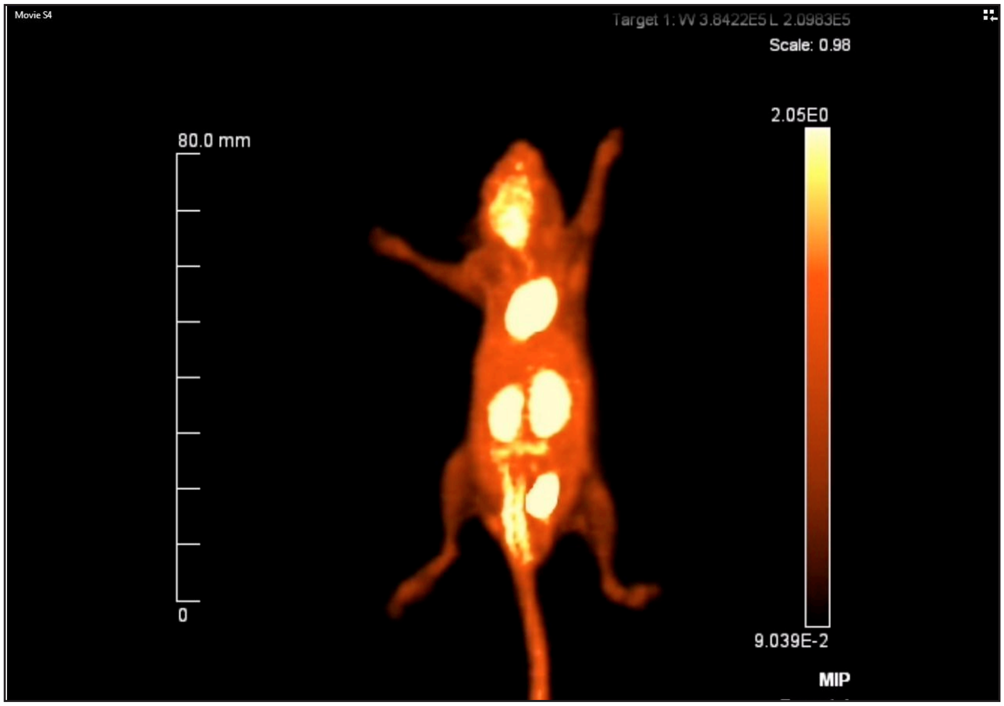

Supplementary Video S4: PET-CT Video of mice in 6 mg/kg arenobufagin group, Related to Figure 8.
